# Ascl1 and Ngn2 convert mouse embryonic stem cells to neurons via functionally distinct paths

**DOI:** 10.1038/s41467-023-40803-y

**Published:** 2023-09-02

**Authors:** Gintautas Vainorius, Maria Novatchkova, Georg Michlits, Juliane Christina Baar, Cecilia Raupach, Joonsun Lee, Ramesh Yelagandula, Marius Wernig, Ulrich Elling

**Affiliations:** 1 Institute of Molecular Biotechnology of the Austrian Academy of Science (IMBA), Dr. Bohr-Gasse 3, Vienna BioCenter (VBC), 1030 Vienna, Austria; 2 Vienna Biocenter PhD Program, a Doctoral School of the University of Vienna and Medical University of Vienna, A-1030 Vienna, Austria; 3 Research Institute of Molecular Pathology (IMP), Campus-Vienna-BioCenter 1, Vienna BioCenter (VBC), 1030 Vienna, Austria; 4 Institute for Stem Cell Biology and Regenerative Medicine, Department of Pathology, Stanford University, Stanford, CA USA; 5 Present Address: JLP Health GmbH, Himmelhofgasse 62, 1130 Vienna, Austria; 6 Present Address: Laboratory of Epigenetics, Cell Fate & Disease, Centre for DNA Fingerprinting and Diagnostics (CDFD), Uppal, Hyderabad, 500039 India

**Keywords:** Reprogramming, Transdifferentiation, Functional genomics

## Abstract

Ascl1 and Ngn2, closely related proneural transcription factors, are able to convert mouse embryonic stem cells into induced neurons. Despite their similarities, these factors elicit only partially overlapping transcriptional programs, and it remains unknown whether cells are converted via distinct mechanisms. Here we show that Ascl1 and Ngn2 induce mutually exclusive side populations by binding and activating distinct lineage drivers. Furthermore, Ascl1 rapidly dismantles the pluripotency network and installs neuronal and trophoblast cell fates, while Ngn2 generates a neural stem cell-like intermediate supported by incomplete shutdown of the pluripotency network. Using CRISPR-Cas9 knockout screening, we find that Ascl1 relies more on factors regulating pluripotency and the cell cycle, such as Tcf7l1. In the absence of Tcf7l1, Ascl1 still represses core pluripotency genes but fails to exit the cell cycle. However, overexpression of Cdkn1c induces cell cycle exit and restores the generation of neurons. These findings highlight that cell type conversion can occur through two distinct mechanistic paths, even when induced by closely related transcription factors.

## Abstract (web summary)

Expression of transcription factors can convert one cell type to another beyond developmental paths. Here, the authors show that cells can take two mechanistically distinct paths in the same transition paradigm when driven by the similar proneural factors Ascl1 and Ngn2.

## Introduction

Cell identity in development is typically crafted by an interplay of multiple transcriptional activators and inhibitors successively restricting the lineage. Cells gradually rewire their gene transcriptional network, retarget multiple transcriptional factors to new loci, reshape their epigenetic landscape, and establish epigenetic barriers to lock the next developmental state^1–5^. In contrast, directed lineage conversions rely on the expression of transcription factors (TF) in alien cellular contexts that can be affected by different distributions of epigenetic marks^6,7^, posttranslational modifications^8,9^, interaction partners^10–13^, and other factors^14,15^. This can affect the binding and activity of the TF. Conflicting or incomplete cues of lineage formation allow the formation of alternative lineages during directed lineage conversion regimes^16–19^. These conversions do not necessarily follow developmental trajectories, and can also skip intermediate cell states^20,21^. As such, they provide an opportunity to study alternative inroads to cell states. Thus, cellular reprogramming can be used as a powerful tool to address fundamental principles underlying lineage specifications and generate models for a study of various diseases as well as potential therapeutic modalities^22,23^.

Basic helix loop helix (bHLH) TFs have proven to be efficient factors for lineage conversion. Various proneural factors, such as Ascl1, Ngn1, Ngn2, Neurod1 and Neurod4 have been utilized to convert different cell types to functioning neurons^24–28^. Among those, Ascl1 and Ngn2 (encoded by the *Neurog2* gene) have emerged as preferred tools for in vitro neuronal reprogramming^24^. Both are master regulators of neuronal fate in the developing central nervous system. Expression of Ascl1 in ventral telencephalon progenitor cells generates inhibitory GABAergic interneurons, whereas Ngn2 directs dorsally situated progenitors toward excitatory neurons with glutamatergic identity. Although, Ascl1 and Ngn2 give rise to different subtypes in the telencephalon, expression of Ngn2 in the ventral telencephalon can rescue Ascl1 null mice^29^. Interestingly, while expression of Ascl1 and Ngn2 in astrocytes can recapitulate developmental neuronal subtype specification to GABAergic or glutamatergic neurons, respectively, expression of Ascl1 in mouse embryonic fibroblasts (MEF), as well as expression of Ascl1 or Ngn2 in mouse embryonic stem cells (ESC), lead to primarily glutamatergic neurons^27,28,30^. Thus, the subtype specification depends on the initial cell type. It is thus vital to understand how ectopically expressed TFs interact with the initial cellular context to define the outcome of conversions.

The ability to transition toward the same states using different transcription factors gives an opportunity to directly contrast differentiation mechanisms. In an elegant study, Aydin et al. showed that Ascl1 and Ngn2 in ESC prefer binding distinct E-box motifs^6^. This, in turn, initiates different accessibility and gene expression patterns, influencing downstream TFs, while still resulting in the formation of glutamatergic induced neurons (iN) in both cases^6,27,28,30^. However, downstream mechanistic differences, such as the downregulation of the initial pluripotency network, are still not understood. Furthermore, even though Ascl1 and Ngn2 possess ‘pioneering’ TF properties, their binding and expression can be influenced by cellular context, e.g., Ascl1 binds and induces muscle lineage when expressed in MEF, but myoblast induction was not reported in ESC^6,16,19^.

In this work, we study the mechanistic differences in how the expression of Ascl1 or Ngn2 transitions cells between two identical states. We observe different side lineages forming in parallel to neurons due to differential binding and different strategies for exiting pluripotency upon Ascl1 and Ngn2 induction: Ascl1 rapidly shuts down the pluripotency network and arrests the cell cycle to install neuronal or trophoblast states, while Ngn2 retains *Sox2* expression to produce neuron stem cells (NSC). CRISPR/Cas9 forward genetic screens revealed that genes involved in pluripotency regulation and cell cycle control are affecting neuronal reprogramming by Ascl1, but not Ngn2. Our results highlight different mechanistic pathways to iN employed by these bHLH TFs.

## Results

### Ascl1 and Ngn2 convert ESC to iN but generate different side lineages

**Fig. 1:**
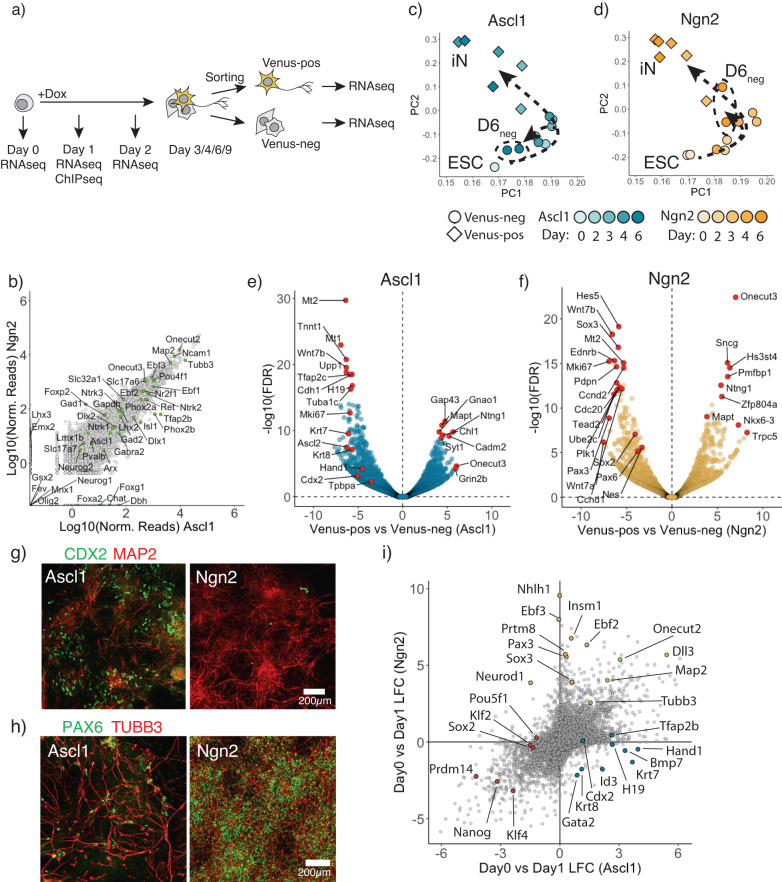
Ascl1 and Ngn2 induce different alternative lineages. **a** Schematic overview of the experimental design. **b** Scatter plot comparing gene expression at Day 6 between Ascl1 and Ngn2 Venus-positive cells with various neuronal subtype specific markers indicated in green. **c**, **d** Principal component analysis of time-resolved bulk RNAseq after Ascl1 (**c**) or Ngn2 (**d**). Each data point corresponds to the single time point replicate. Color intensity shows day post-induction. Shape corresponds the Mapt-Venus reporter upregulation. Arrows show the trajectory cells take after the Ascl1 (**c**) or Ngn2 induction (**d**). **e**, **f** Vulcano plot comparing gene expression between Venus-positive and negative populations at day 6 post-induction of Ascl1 (**e**) or Ngn2 (**f**). Red circles denote top significantly upregulated or downregulated genes as well as example genes marking in trophoblast (**e**) or NSC lineages (**f**). **g** Representative immunostained cells for a trophoblast marker CDX2 and a neuronal marker Map2 at day 6 post-induction of Ascl1 or Ngn2. Trophoblast markers were expressed only after Ascl1 induction, but not Ngn2. **h** Representative immunostained cells for an NSC marker PAX6 and a neuronal marker TUBB3 at day 6 post-induction of Ascl1 or Ngn2. NSC markers were expressed only after Ngn2 expression, but not Ascl1. **i** Scatter plot comparing gene expression changes between Ascl1 and Ngn2 at day 1 post-induction. Highlighted circles are example genes that are neuronal markers expressed in both (green), trophoblast Ascl1 specific markers (blue), NSC Ngn2 specific markers (yellow), pluripotency related genes (red). Source data are provided as a Source Data file.

Ectopic expression of Ascl1 or Ngn2 in mouse embryonic stem cells (ESCs) is sufficient to induce terminal differentiation into neurons^28^. Yet, the differences in transition mechanism toward neurons as well as possible side populations are not well characterized^6,31^. To examine this cell type conversion in detail, we generated clonal ESC cell lines expressing rtTA and TetO-Ascl1 or TetO-Ngn2^27,28^ (Supplementary Fig. 1a). After doxycycline (Dox) addition, ESCs are rapidly converted to induced neurons: Ascl1 and Ngn2 produce cells expressing the neuronal marker TUBB3 and displaying neuronal morphology from day 3 and day 2 onward, respectively (Supplementary Fig. 1a). To report neuronal fate in these cell lines we endogenously tagged the pan-neuronal marker gene Mapt on its C-terminus with the fluorescent protein Venus^27^ (Supplementary Fig. 1b) and performed time-resolved bulk RNAseq upon Dox-induction. Cells were sorted into Venus-positive neurons and Venus-negative cell populations (Fig. 1a) from day 3 onward. As reported before^28,30^, both Ascl1 and Ngn2 give rise to similar iN cell identities (Fig. 1b; Supplementary Fig. 1c–e). Thus, initial ESC and terminal iN states are very similar between Ascl1 and Ngn2-induced conversions (Supplementary Fig. 1b bottom). This is in line with previous observations that transcriptomes converge to drive iN formation despite differences in the initial transcriptional response^6^ and follow an overall similar trajectory between ESC and iN in the PCA analysis (Fig. 1c, d; Supplementary Fig. 1g).

To investigate cells that fail to make iN in more detail, we focused on the Mapt-Venus-negative cells, which could represent incomplete or alternative differentiation outcomes. Mapt-Venus-negative cells generated by Ascl1 cluster closer to the initial ESC populations in PCA plots than Ngn2-induced Mapt-negative cells (Fig. 1c). However, this is not due to retaining a population of undifferentiated ESC as only a marginal number of cells express ESC marker NANOG in terminal population (Supplementary Fig. 1g), and NANOG and OCT4 are not expressed in Mapt-Venus population (Supplementary Fig. 1h). To identify the alternative type of cells generated by Ascl1, we used PanglaoDB^32^ using genes differentially expressed between Venus-negative and positive populations at day 6 (Fig. 1e, f; Supplementary Fig. 2a). Interestingly, Ascl1 produce cells expressing trophoblast markers such as *Hand1*, *Cdx2*, *Tpbpa*, *Krt8* (Fig. 1e, g; Supplementary Fig. 2b–g) with mesenchymal morphology, which were not present in Ngn2-induced cultures (Fig. 1g, Supplementary Fig. 2b, c, f). We termed these cells induced-Trophoblast-like-cells (iT). Interestingly, many of the *Krt8*, *Cdx2* positive iTs appeared binucleated, which could be a result of multinucleation similar to trophoblast lineage development in vivo (Supplementary Fig. 2e)^33^. The induction of iT could be due to Ascl1 mimicking the bHLH transcription factor Ascl2, a driver for the trophoblast lineage. Both Ascl1 and Ascl2 are evolutionary close and share near identical DNA binding domains and bind similar E-box motifs (Supplementary Fig. 2h–k)^33–37^. Lastly, to exclude clonal effects of the cell line used, we repeated these experiments in the background of an alternative mouse ESC line, E14. We introduced rtTA via a piggybac transposon vector and expressed Ascl1 from a Dox-inducible viral vector^27^ and generated 24 single-cell derived clones. All the clones showed the formation of both iN and iT, suggesting that the formation of iTs is a reproducible side product of ectopic Ascl1 expression in mouse ESCs (Supplementary Fig. 3).

In contrast to Ascl1 induction, Ngn2 induces Mapt-negative cells expressing NSC markers, such as *Sox2*, *Pax3*, *Pax6*, *Nes* (Fig. 1f, h; Supplementary Fig. 2a, 4a, b), as previously described^21,38^. Furthermore, Ngn2 reprogramming could be locked in the NSC-like state (iNSC) in the presence of FGF2 and EGF and is dependent on the Notch pathway^39,40^ (Supplementary Fig. 4c, d). In contrast, we did not observe NSC markers upregulated during Ascl1-induced differentiation (Fig. 1h, Supplementary Fig. 4b, c). To see if Ngn2 can use iNSC state as proliferative intermediate, we differentiated cells in the presence or absence of cytosine β-D-arabinofuranoside (AraC) from day 4 post-induction to inactivate dividing cells (Supplementary Fig. 4e). Indeed, addition of AraC drastically reduces Ngn2-produced iNs, while Ascl1 was insensitive to AraC treatment, suggesting that no continuously proliferative intermediate is present during Ascl1-induced iN reprogramming (Supplementary Fig. 4e). In summary, despite Ascl1 and Ngn2 converting mESC to similar iN subtypes, Ascl1 and Ngn2 produce distinct additional alternative cell lineages, suggesting that despite identical initial and terminal populations, differences exist that we sought to understand further (Supplementary Fig. 1b bottom).

### Ascl1 and Ngn2 initiate paths with different transcriptional programs

To get a better understanding of different transcriptional response invoked by Ascl1 and Ngn2, we performed bulk RNAseq and ChiPseq on day 1 (Fig. 1a). Both Ascl1 and Ngn2 induce general neuronal markers such as *Tubb3*, *Map2* and *Onecut2* and downregulate general pluripotency markers like *Nanog*, *Klf4* (Fig. 1i). Furthermore, Ascl1 strongly induces downstream targets *Tfap2b*, *Lmx1b*, while Ngn2 strongly upregulates *Neurod1*, *Nhlh1* (Fig. 1i). In addition, Ascl1 upregulate Trophoblast lineage markers, e.g., *Krt7/8*, *Hand1*, while Ngn2 upregulates expression of NSC related genes, like *Pax3* and *Sox3* (Fig. 1i). Interestingly we observed that early in reprogramming cells are positive for both—neuronal and trophoblast markers (Supplementary Fig. 5a, b). In addition, we reanalyzed available scRNAseq data^6^ for Day 2 of ESC to iN conversion by Ascl1 and Ngn2 and could also observe cells positive for both neuronal and trophoblast markers (Supplementary Fig. 5c, d). This suggests that Ascl1 can induce both lineages simultaneously, which later are resolved into iN or iT cells (Supplementary Fig. 5b).

As reported by Aydin and colleagues^6^, Ascl1 and Ngn2 show different preferences for E-box motives, which in turn result in the activation of different subset of genes. Indeed, we confirmed Ascl1 and Ngn2 differential binding in ESC (Supplementary Fig. 6a). Furthermore, we see that Ascl1 and Ngn2 target genes differentially expressed between them as well as genes involved in the different alternative lineages, e.g., *Hand1*, *Cdx2*, *Krt8* or *Neurod1*, *Pax3*, respectively (Supplementary Fig. 6b). Interestingly, Ascl1 binds trophoblast related genes also in MEF, although without iT induction (Supplementary Fig. 7a, b). However, in addition to iN induction, Ascl1 overexpression in MEF leads to Ascl1 binding to the skeletal muscle genes and the induction of myocytes^7,16^. Indeed, in ESC Ascl1 also strongly bind to skeletal muscle lineage-related genes, e.g., *Myod1*, *Myog*, *Myf3*, *Tnnt2* (Supplementary Fig. 7c). However, we did not observe upregulation of these genes (Supplementary Fig. 7d). Thus, it is tempting to speculate that cellular context, such as cell type-specific histone modifications or transcription factors, affects the choice of the alternative lineage induced. Thus, in addition to the induction of the neuronal transcriptional program, additional genes are bound and transcribed, leading to the formation of alternative lineages. This “off target” transcriptional program depends on overexpression of transgene, e.g., Ascl1 or Ngn2, as well as the cellular context, e.g., ESC or MEF.

To investigate the general principles of the early transcriptional response in more depth, we used the STRING database to examine the gene regulatory network (GRN) of Ascl1 and Ngn2-upregulated differentially expressed genes (DEGs) one day after induction (Supplementary Fig. 8a, b). Both Ascl1 and Ngn2 DEGs form networks containing three distinct gene groups. Interestingly, Ascl1 and Ngn2 GRNs share groups containing genes involved in RNA and sterol metabolism, which can be attributed to the metabolic shift during conversion^41,42^. Furthermore, Ngn2 forms a more interconnected network than Ascl1, suggesting that Ngn2 invokes a more coherent transcriptional response than Ascl1 (Supplementary Fig. 8c).

To analyze the differences in more detail, we looked at the central-most connected nodes of both GRNs (Supplementary Fig. 8d, e). In contrast to Ascl1, Ngn2 induce genes driving neuronal differentiation *Neurog2*, *Neurod1*, *Neurog1*, *Lhx2/3*, *Otx2*, as well as genes involved in neural stem cell differentiation: *Notch1*, *Hes5*, *Pax3*, centering GRN around them (Supplementary Fig. 8e–g). Interestingly, Ngn2, Neurod1 and Ngn2 are known to be able to convert ESC to iN^43^. Thus, such a positive feedback loop together with the induction of strong lineage drivers and highly interconnected GRN can allow a more robust iN conversion and faster independence from the initial induction of the cassette. To test this hypothesis, we induced cells for 2, 4, or 6 days (Supplementary Fig. 8h). Indeed, we observe that efficient iN conversion with Ascl1 relies on the sustained expression of Ascl1, while Ngn2 efficiently induces conversion already after 2 days of induction (Supplementary Fig. 8h).

In summary, Ascl1 and Ngn2 bind and invoke different transcriptional profiles and subsequent mechanistic differences of the ESC to iN conversion. Where Ascl1 induction relies on the sustained expression of Ascl1, Ngn2 induces an overall more coherent network allowing efficient and fast reprogramming as well as induction of a proliferative intermediate.

### Loss-of-function CRISPR/Cas9 screen to identify genetic dependencies for ESC to iN conversion

**Fig. 2:**
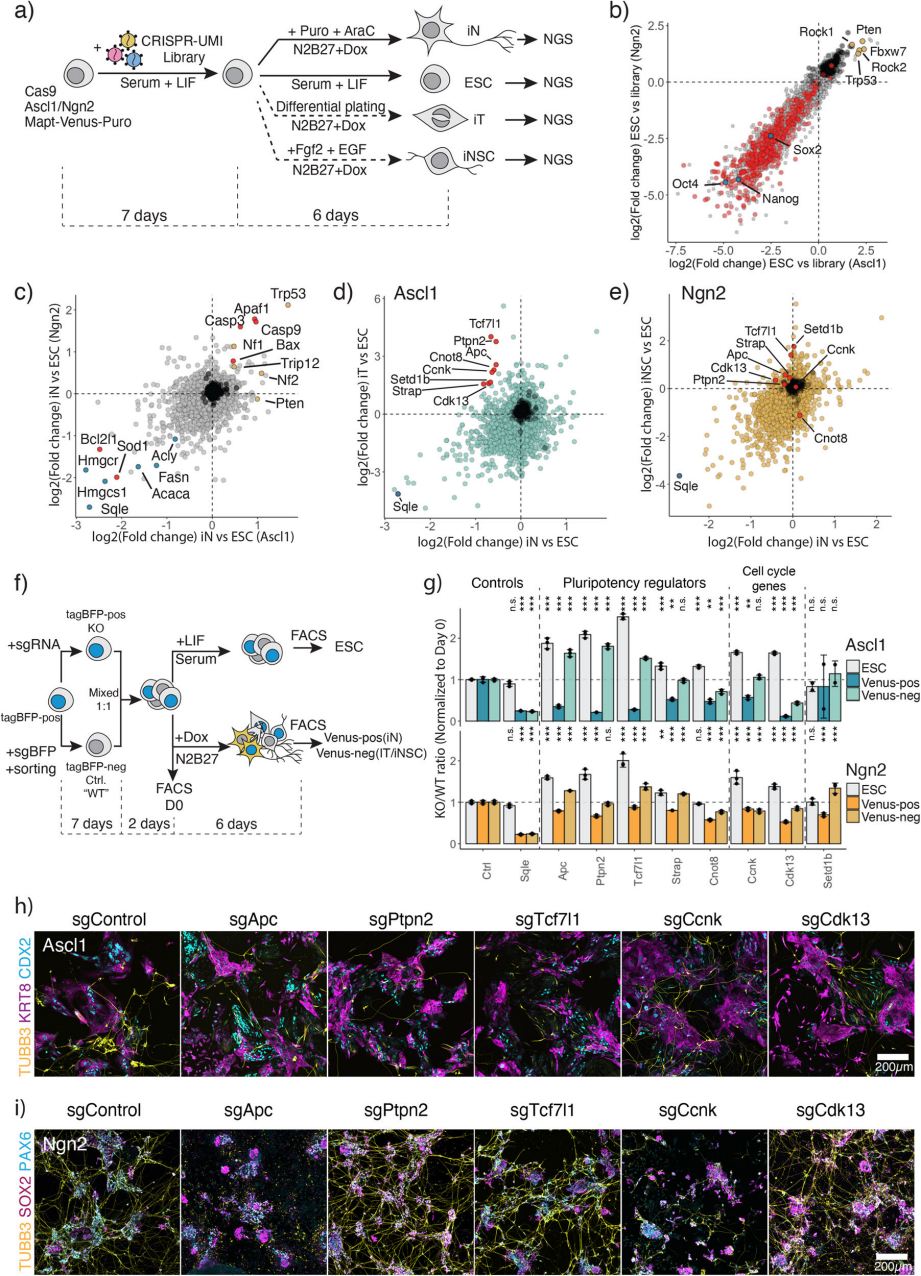
CRISPR-Cas9 forward genetic screen to identify genetic dependencies of forward differentiation by Ascl1 or Ngn2. **a** CRISPR-Cas9 screen experimental outline. **b** Comparative analysis of the gene knockout effects between Ascl1 and Ngn2 transgenes carrying ESC. Difference in guide abundance was calculated between uninduced ESC at Day 13 post library infection (**a**) versus library plasmid pool. Dots represent genes; axis shows depletion in LFC in each cell line. Red represents core essential genes as defined by Hart et al.^45^, blue—core pluripotency genes, yellow—tumor suppressors. **c** Comparative analysis of gene knockout effects of Ascl1 or Ngn2-induced iN versus ESC. Red dots represent apoptosis related genes, yellow—tumor suppressor genes, blue—cholesterol biosynthesis genes. **d** Comparative analysis of gene knockout effects of Ascl1-induced iN or iT versus ESC. Red dots represent genes chosen for validation. Sqle is a positive control for a strong depletion. **e** Comparative analysis of gene knockout effects of Ngn2-induced iN or iNSC versus ESC. Red dots represent genes chosen for validation in (**d**). Sqle is a positive control for a strong depletion. **f** Experimental outline of hits validation from (**d**). **g** FACS-based validation of the hits. Bar represents the normalized ratio to the initial mixture ratio at day 0. *N* = 3 independent biological replicates. Bar plot shows mean ± SD. *p*-values, indicated above, were determined by one-way ANOVA followed by Dunnett’s multiple comparison test (two-sided) using Ctrl ratio as a control. “n.s.” not significant, “*” *p* < 0.05, “**” *p* < 0.01, “***” *p* < 0.001. **h**, **i** Immunostaining of the knockout cells for neuronal marker Tubb3 and alternative lineage markers KRT8/CDX2 for Ascl1-induced iT (**h**), and SOX2/PAX6 for Ngn2-induced iNSC (**i**). Source data are provided as a Source Data file.

To establish a mechanistic handle on the underlying differences between Ascl1- and Ngn2-induced ESC reprogramming, we performed a CRISPR/Cas9 loss-of-function screen. We aimed to investigate two aspects of this conversion: first, the genetic dependencies of Ascl1 and Ngn2-induced neuronal conversion, and second, to elucidate the genes involved in alternative state formation (Fig. 2a). For this, ESC were infected with retroviral CRISPR-UMI sgRNA library containing ~27,000 guides targeting 6630 genes (4 guides per gene) and 108 non-targeting guides^44^. We then differentiated ESC for 6 days and enriched for iN using AraC and puromycin treatment (Fig. 2a, Supplementary Fig. 1b, 9a). In addition, we assessed dependencies for iT and iNSC differentiation (Fig. 2a, see “Methods”). For an initial assessment of the screening results, we compared the depletion of known essentials in ESC versus the Library (Fig. 2b). We indeed observed a strong depletion in common essential genes as defined by Hart et al.^45^. Furthermore, genes at the core pluripotency network (*Nanog*, *Sox2*, *Pou5f1*) are among the most essential genes, while tumor suppressors including *Trp53*, *Fbxw7*, *Rock1* are strongly enriching in ESC (Fig. 2b), showing that our library is effective at gene targeting and revealing gene knockout phenotypes.

To identify common genetic dependencies of iN formation, we compared guide abundance in the ESC versus iN at D6 (Fig. 2c). Guides showing the strongest depletion are targeting, for instance, fatty acid metabolism and sterol biosynthesis, such as *Fasn*, *Hmgcr*, *Hmgcs1*, *Sqle*, an essential component for neuronal metabolism (Fig. 2c)^41,42^. Furthermore, during iN differentiation, cells undergo high levels of stress due to lipid peroxidation and can commit cell death via apoptosis or ferroptosis^46,47^. Hence, we find proapoptotic genes, *Casp3*/*9* and *Bax* enriching, while antiapoptotic genes such as *Bcl2l1* and *Sod1* are depleting from the population (Fig. 2c; Supplementary Fig. 9b). In addition, knockout of tumor suppressors, e.g., *Trp53*, *Nf1*, *Nf2*, allows for higher iN conversion. Thus, this screen yields genetic dependencies in ESC to iN conversion at high resolution.

To uncover differential genetic dependencies between iN and alternative states, relative sgRNA abundance was correlated with a particular focus on genes depleted in iN and enriched in the alternative states. We focused on differential dependencies between the Ascl1-induced iN and iT (Fig. 2d). We picked genes for validation based on the following criteria: (1) genes showing little effects in ESC (−2 < LFC < 2); (2) genes showing enrichment in iT (LFC > 1.5); (3) genes depleted in iN (LFC < −0.5). We noticed that genes important in the regulation of pluripotency network and differentiation of ESC: *Tcf7l1*, *Ptpn2*, *Apc*, *Strap*, *Cnot8*^48–54^, as well as cell cycle genes *Ccnk* and *Cdk13*^55,56^ are required for Ascl1-induced iN conversion while inhibiting iT formation. In contrast, knockout of these hits had little to no influence on Ngn2-driven conversion to neurons (Fig. 2d, e).

To validate the findings, we performed a competition assay. To implement an internal control, we introduced a constitutive tagBFP vector in our cell line and derived a clone with a stable tagBFP expression. The cells were then infected with the sgRNA that showed the strongest depletion in the screen. Separately, we generated an isogenic control population, by targeting tagBFP with a control guide against tagBFP and sorting for tagBFP^neg^ cells. Subsequently, knockout and control cells were mixed in a 1:1 ratio and maintained for an additional passage to adapt them to the same conditions, before plating cells for induction (Fig. 2f). After 6 days of induction, we assessed iN Mapt-Venus-positive population, as well as Venus-negative population, corresponding to the formation of iT/iNSC populations (Fig. 2f, g). In addition, we assessed the fitness of the knockouts in the ESC cells while growing ESC for additional 6 days (Fig. 2f). As anticipated, *Sqle* was essential in iN, iT or iNSC populations, while not in ESC cells, mirroring the screen results (Fig. 2c–e, g). Similarly, knockout of pluripotency-related genes *Apc*, *Tcf7l1* and *Ptpn2* abolished the formation of iNs upon induction with Ascl1, while allowing the formation of iT cells (Fig. 2g). We further confirmed the phenotype via immunostaining at day 6 post-induction (Fig. 2h, i). Knockout of the hits resulted in failure to generate TUBB3 positive neurons by Ascl1 while still generating CDX2+ and KRT8+ cells. Interestingly, some cells expressing neuron-specific TUBB3 lack neuronal morphology, indicating activation of neuronal genes but failure to establish a final iN state (Supplementary Fig. 9c). Likewise, targeting of *Ccnk* or *Cdk13* strongly reduced the number of iNs upon Ascl1 induction. In contrast to Ascl1, Ngn2 iN conversion is not impaired upon loss of *Tcf7l1*, *Ptpn2*, and *Cdk13* and Ngn2 can still generate both neurons and neural stem cells, marked by SOX2 and PAX6 (Fig. 2g–i). However, *Apc* and *Ccnk* knockouts did affect the formation of iN and iNSC induced by Ngn2. Off note, we additionally validated multiple other genes showing differential dependencies between Ascl1 and Ngn2 induction (Supplementary Fig. 9d–h). Taken together, our parallel CRISPR/Cas9 loss-of-function screens unveiled multiple common and differential dependencies between Ascl1- and Ngn2-induced directed differentiation to iNs.

### Rapid downregulation of pluripotency network upon Ascl1 induction

As the screen identified genes involved in maintaining the pluripotency network as essential for the Ascl1-induced iN formation, we investigated how Ascl1 and Ngn2 disassemble the pluripotency network in more depth. For this, we used ingenuity pathway analysis using all differential expressed genes one day post Ascl1- or Ngn2-induction (Supplementary Fig. 10a–d, Fig. 1i). As before, ingenuity canonical pathway enrichment analysis showed that both Ascl1 and Ngn2 induce pathways related to cholesterol biosynthesis (Supplementary Fig. 10b, d). Interestingly, the Ascl1 transcriptional response is centered around the downregulation of the ESC pluripotency network and self-renewal (Supplementary Fig. 10a, b).

**Fig. 3:**
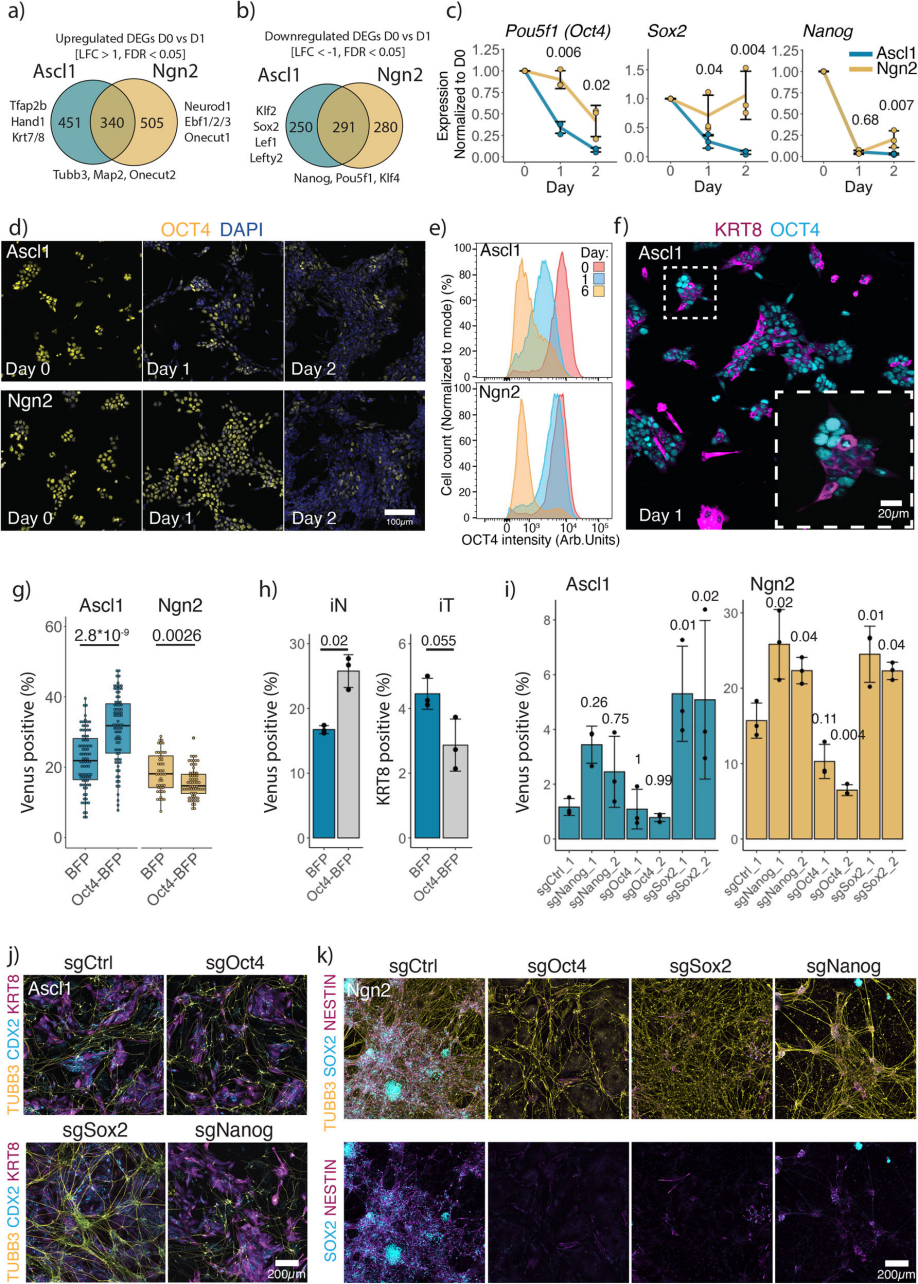
Dynamics of pluripotency network shutdown. **a**, **b** Number of Ascl1, Ngn2 specific or common upregulated (**a**) and downregulated (**b**) genes from Fig. 1i. **c** qPCR data of the expression of the core pluripotency genes (normalized to the expression of Actin and day 0). Lines are drawn through the mean of *n* = 3 biologically independent samples; error bars indicate ± SD. Above, *p*-values of the two-sided Welch two-sample t-test comparing PPN genes expressed between Ascl1 and Ngn2 at the given time point. **d** Representative immunostainings for the OCT4 dynamics after Ascl1 or Ngn2 induction. **e** Quantification of OCT4 expression using intracellular immunostaining followed by FACS. **f** Representative immunostaining for the pluripotency marker OCT4 and trophoblast marker KRT8 at day 1 after Ascl1 or Ngn2 induction. **g** Efficiency of iN formation in the presence of OCT4 overexpression. Each dot represents an individual ESC clone containing an overexpression construct. Efficiency is measured by the percentage of Mapt-Venus population. Boxplots indicate 25th and 75th percentiles as bounds of the box with the median center line; whiskers indicate minima/maxima of a 1.5x distance of the IQR from the 25th and 75th percentiles. *p*-value of the two-sided Welch two-sample t-test indicated above. **h** Efficiency of iN formation, measured by the percentage of Mapt-Venus expressing cells, and iT formation, percentage of cells immunostained for KRT8 of polyclonal population overexpressing Oct4-BFP. Bar plot shows mean of *n* = 3 independent biological replicates with ± SD; the *p*-values were calculated using the two-sided Welch two-sample t-test. **i** Efficiency of iN formation upon acute knockout of core pluripotency. Efficiency is measured by the percentage of Mapt-Venus population. The bar plot shows mean of *n* = 3 biologically independent samples with ± SD. *p*-values, indicated above, were calculated using one-way ANOVA followed by Dunnett’s multiple comparison test (two-sided) using sgCtrl as a control. **j** Representative immunostainings of (**i**) for iN and iT induced by Ascl1. **j** Representative immunostainings of (**i**) for iN and iNSC induced by Ngn2. Source data are provided as a Source Data file.

We then divided DEGs into Ascl1 or Ngn2 specific, or common and performed KEGG pathway enrichment analysis (Fig. 3a, b; Supplementary Fig. 10e). Interestingly, Ascl1 upregulates genes involved in cellular senescence and cell cycle exit, such as *Cdkn1a, Cebpa*, *Cebpb* (Supplementary Fig. 8d, g)^57,58^, as well as downregulates more genes involved in pluripotency, e.g., *Pou5f1* (encoding for protein OCT4), *Klf2*, *Sox2*, *Lef1*, *Lefty2*, compared to Ngn2 (Fig. 3b, Fig. 1i, Supplementary Fig. 10e). Thus, we next looked at the dynamics of the pluripotency network (PPN) shutdown upon Ascl1 and Ngn2 induction. For this, we focused on the three core pluripotency genes: *Pou5f1* (Oct4), *Sox2*, *Nanog* (Fig. 3c, Fig. 1i). *Nanog* is downregulated at similar kinetics between Ascl1 and Ngn2 (Fig. 3c, Fig. 1i, Supplementary Fig. 11a, b). In contrast, *Sox2* expression is retained after Ngn2 induction as an NSC gene regulatory network is established, while Ascl1-induced cells lose expression of *Sox2* (Fig. 3c, Fig. 1i, Supplementary Fig. 11a, b). Similarly, Ascl1 induction leads to a rapid loss of Oct4 expression, while Ngn2 induction leads to gradual downregulation of Oct4 (Fig. 3c–e, Fig. 1i). Furthermore, loss of Oct4 corresponds with the upregulation of trophoblast marker KRT8 (Fig. 3f).

To test the functional relevance of the loss of Oct4, we constitutively overexpressed Oct4 and generated multiple clonal mESC cell lines (Fig. 3g, h). While overexpression of Oct4 together with Ngn2 leads to a reduction of the iN population, co-expression of Oct4 and Ascl1 increases the efficiency of iN formation (Fig. 3g, h). In turn, iT formation is hampered in the presence of Oct4 (Fig. 3h). During development, Oct4 inhibits differentiation of the trophectoderm lineage, and rapid loss of Oct4 is associated with the upregulation of the trophectoderm markers^59,60^. Thus, rapid loss of Oct4 generates permissive conditions for iT lineage formation, that compete with the formation of iN.

Given the kinetic differences in the downregulation of PPN after Ascl1 or Ngn2 induction, we tested if enforced disruption of the PPN together with differentiation would affect the ESC to iN conversion. As *Pou5f1* (Oct4), *Sox2* and *Nanog* are essential for ESC (Fig. 2b), we infected cells with the guides targeting *Pou5f1* (Oct4), *Sox2* and *Nanog* 2 days before the induction of Ascl1 or Ngn2 (Fig. 3i–k). Interestingly, Ascl1 induction leads to the formation of both iN and iT in the knockout of all pluripotency factors (Fig. 3j). We see little to no effect after targeting Oct4, as Oct4 is rapidly lost upon Ascl1 induction (Fig. 3i). However, knockout of Nanog or *Sox2* favors iN formation, suggesting that additional disruption of the PPN supports installment of the iN state (Fig. 3i, j). In turn, knockout of *Sox2* abolished the formation of iNSC by Ngn2, showing that *Sox2* is repurposed from the PPN to the NSC gene regulatory network (Fig. 3i, k). Similarly, disruption of either *Nanog* or *Pou5f1* (Oct4) also resulted in loss of iNSC (Fig. 3k). Instead, we observed formation of primitive endoderm and trophoblast upon loss of *Nanog* or *Pou5f1* (Oct4), respectively (Fig. 3k, Supplementary Fig. 11c, d), in alignment with the outcome of the loss of these factors during the development^59–61^.

This data, together with differential dependencies identified in the CRISPR screen, shows that Ascl1 and Ngn2 induction leads to pronounced functional differences in exiting the pluripotency state of ESC. Ascl1 induction leads to an efficient shutting down of PPN and later induction of iN or iT gene network, while Ngn2 does not fully downregulate the PPN and instead overlays both networks, repurposing genes for the induction of NSCs.

### Tcf7l1 is required for cell cycle exit to generate Ascl1 iNs

We next focused on Ascl1-induced PPN shutdown. In Ascl1-driven MEF to iN transdifferentiation, Myt1l facilitates iN formation by repressing initial fibroblast GRN as well as alternative myoblast lineage^16,62^. Given the fast downregulation of PPN, we tested if Myt1l is similarly required for ESC to iN conversion. However, our screen did not reveal that *Myt1l*, or its paralogs *Myt1* and *St18*, are required for ESC to iN conversion (Supplementary Fig. 12a) as well as validation experiments using additional sgRNAs (Supplementary Fig. 12b). In the CRISPR screen we identified *Tcf7l1* (T cell factor/lymphoid enhancer factor, also known as Tcf3), a repressor of PPN, as essential for iN formation by Ascl1. We confirmed the *Tcf7l1* knockout phenotype in a separate E14 background and ruled out that the loss of Tcf7l1 affected the expression of the Ascl1 transgene (Supplementary Fig. 12c–e). Tcf7l1 is poised on the multiple promoters of PPN-related genes and rapidly represses their expression upon ESC differentiation^48,63,64^, and the absence of Tcf7l1 stabilizes the ESC state^64^. Thus, we wanted to test if Tcf7l1 is required before or after the onset of cell type conversion, as well as exclude secondary effects due to the stabilization of the PPN before the conversion. For this, we N-terminally tagged Tcf7l1 with an Auxin inducible degron, infected cells with lentiviral vector carrying osTIR1 (F-box E3-ubiquitin ligase, derived from Oryza sativa) and generated single cell-derived clones (Supplementary Fig. 12f)^65^. We then depleted Tcf7l1 before or at the onset of conversion by the addition of Auxin (Supplementary Fig. 12g). While Tcf7l1 depletion before induction had only a mild effect on the formation of iN, degrading Tcf7l1 on the onset of Ascl1 induction severely reduced the number of neurons produced by Ascl1 (Supplementary Fig. 12g). This indicates that Tcf7l1 acts after the induction rather than stabilizing the PPN.

**Fig. 4:**
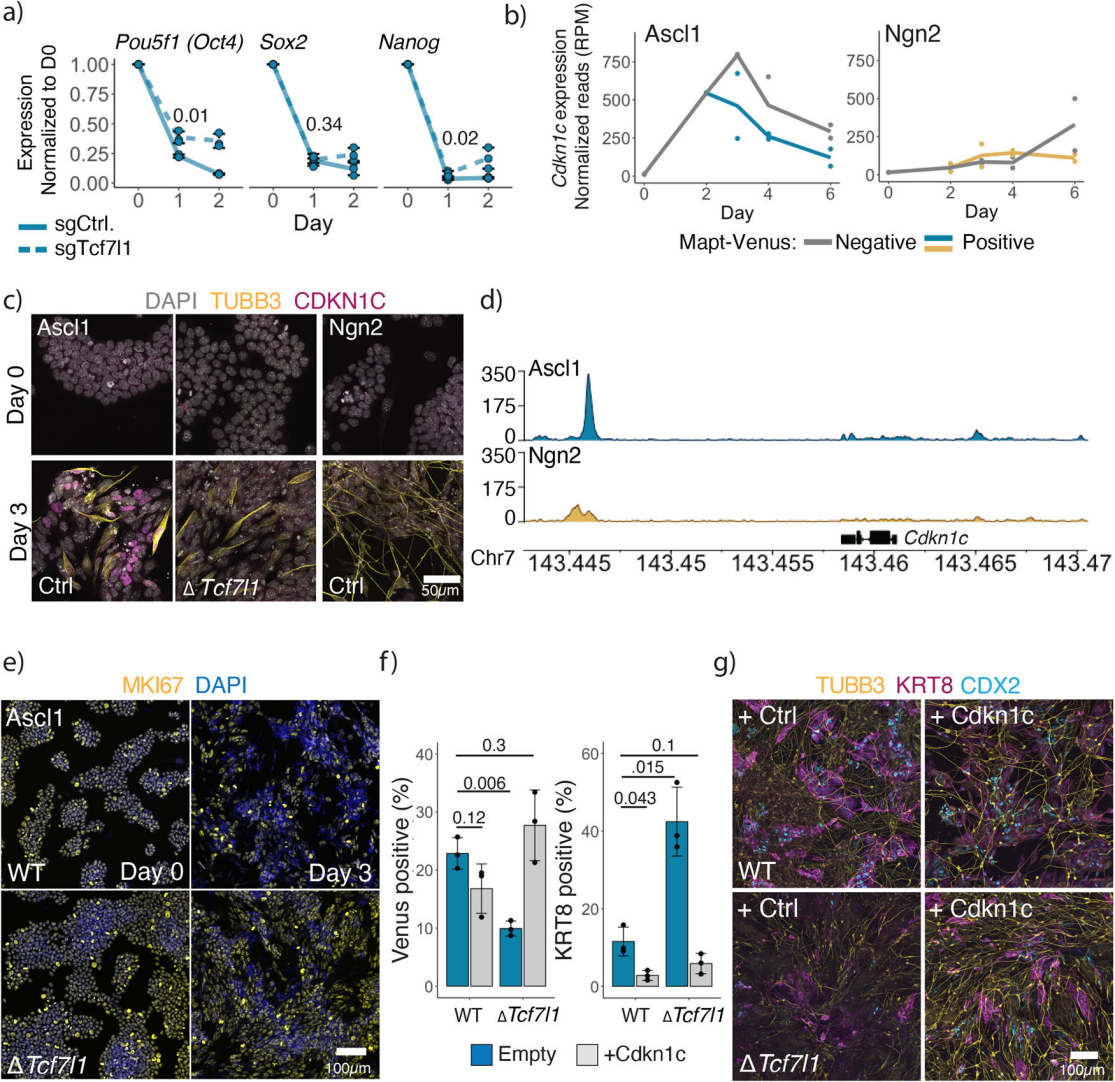
Overexpression of Cdkn1c rescues Tcf7l1 knockout phenotype. **a** qPCR of core pluripotency gene expression after induction in WT and *Tcf7l1* KO cells. Expression normalized to Actin and day 0. Lines are drawn through the mean of *n* = 3 biologically independent samples; error bars indicate ± SD. *p*-values of the two-sided Welch two-sample t-test comparing sgTcf1l1 vs sgControl at day 1 indicated above. **b** *Cdkn1c* expression during ESC to iN conversion (Fig. 1a). **c** Immunostainings of CDKN1C on day 0 and day 3 of the ESC to iN conversion by Ascl1 WT or *Tcf7l1* KO or Ngn2 expressing cells. **d** Binding of the Flag-Ascl1 or Flag-Ngn2 in the *Cdkn1c* locus at day 1 post-induction. Data showing combined reads of four replicates. **e** Immunostainings of proliferation marker MKI67 after induction of Ascl1 in WT or *Tcf7l1* KO ESC cells. **f** FACS data of Cdkn1c overexpression with Ascl1 in WT or *Tcf7l1* KO ESCs. The efficiency of iN formation is measured by the percentage of Mapt-Venus population, efficiency of iT formation is measured by the percentage of cells immunostained for KRT8. The bar plot shows mean of *n* = 3 independent biological replicates with ± SD. *p*-value of the two-sided Welch two-sample t-test indicated above. **g** Representative images of immunostained cells for neuronal TUBB3 and trophoblast CDX2/KRT8 markers at day 6 post-induction of WT or *Tcf7l1* KO ESCs with Ascl1 and Cdkn1c. Source data are provided as a Source Data file.

To understand the role of Tcf7l1 in the Ascl1-induced ESC to iN-directed differentiation, we generated single cell-derived clones with a homozygous *Tcf7l1* knockout and performed bulk RNAseq at day 1 post-induction (Supplementary Fig. 13a). As Tcf7l1 acts as a pluripotency network repressor during ESC differentiation, we first looked at the pluripotency shutdown in the *Tcf7l1* knockout cells. Surprisingly, the PPN was still downregulated after Ascl1 induction in the absence of Tcf7l1 (Fig. 4a; Supplementary Fig. 13a). However, we observed a group of genes that failed to be expressed in *ΔTcf7l1* clones (Supplementary Fig. 13a). Interestingly, *Cdkn1c* is highly upregulated after Ascl1 induction in comparison to *ΔTcf7l1* (Supplementary Fig. 13a), and its expression is specific for Ascl1 induction and peaks at day 3 of ESC to iN conversion (Fig. 4b, c; Supplementary Fig. 13b). In addition, Ascl1 shows strong binding near *Cdkn1c* locus in comparison to Ngn2 (Fig. 4d).

### Ascl1-directed differentiation is cell cycle-dependent

Cdkn1c is a cyclin-dependent kinase inhibitor of the cip/kip family regulating cell cycle arrest in G1^66^. Cdkn1c is essential for embryonic development, and mice lacking Cdkn1c die perinatally with multiple developmental defects. Furthermore, Cdkn1c is important for the development of early placenta to initiate the endoreplication of trophoblasts^66^. Recently, Cdkn1c was also implicated in the suppression of pluripotency in mouse ESC^67^. We furthermore see that Ascl1-induced GRN central nodes contain multiple genes involved in cell cycle regulation, e.g., *Cdkn1a*, *Cdkn1b*, *Cebpa*, *Cebpb* (Supplementary Fig. 8d, g). Thus, we hypothesized that in *ΔTcf7l1* cells, Ascl1 is unable to arrest the cell cycle. Indeed, we observe prolonged expression of MKI67 as well as a higher percentage of dividing cells after induction of Ascl1 in the *ΔTcf7l1* cells (Fig. 4e, Supplementary Fig. 13c). To test if the expression of Cdkn1c is sufficient to induce iN formation in the absence of Tcf7l1, we coexpressed Cdkn1c together with Ascl1. Indeed, we see a partial rescue of iN formation as well as a decrease in iT generation (Fig. 4f, g). Furthermore, we see that expression of Cdkn1c generates a more mature neuronal and trophoblast phenotype also in WT populations by Day 6 (Fig. 4g). Of note, Cdkn1c is not essential for the formation of Ascl1-induced iNs, suggesting that a group of cell cycle regulators, rather than Cdkn1c alone, is responsible for the cell cycle arrest (Supplementary Fig. 13d).

Taken together, our data show that in Ascl1-induced ESC to iN conversion, cell cycle arrest is a roadblock after exiting the pluripotency state and that Cdkn1c is sufficient to overcome this roadblock. In contrast, this dependency is not observed for Ngn2-dependent iN formation, where the cell cycle is maintained and some ESCs transit to NSCs.

## Discussion

Overexpressing proneural bHLH transcription factors Ascl1 or Ngn2 in mouse ESC show several differences in the conversion toward iN: (1) formation of different alternative lineages; (2) early transcriptional changes in downregulation of pluripotency network and upregulation of new transcripts; (3) different genetic dependencies. In addition to iN, Ascl1 induces trophoblast-like cells marked by Krt8 and Cdx2, while Ngn2 invokes an NSC-like state^38^. The induction of alternative states results from the binding and upregulation of alternative lineage specifying factors, e.g., *Hand1*, *Cdx2* in Ascl1 case and *Pax3* in Ngn2. To induce trophoblast lineage, Ascl1 in mESC acts similarly to Ascl2, a key trophoblast lineage driving the bHLH transcription factor. Interestingly, during MEF to iN transdifferentiation, Ascl1 also binds trophoblast lineage drivers in MEF, although without inducing them (Supplementary Fig. 7a, b). Instead, additionally to iN, Ascl1 expressed in MEF induces myocyte-like cells^16,19^. In turn, muscle lineage genes and other targets are bound in both ESC and MEF^16,19^ (Supplementary Fig. 7c, d). Thus, besides the neuronal lineage, only those Ascl1-bound genes are induced, which are developmentally close to the initial cell lineage in which Ascl1 is expressed. Differences in epigenetic landscape, histone modifications around binding targets as well as interaction partners or cofactors present in the initial cell types could specify binding and induction of target genes molding the mechanism^6–9,12,14^. Thus, the iN conversion paradigm from different cell types using the same transcription factor provides a powerful experimental platform to study how transcription factors binding and induction of lineages can be affected by cellular context.

An important aspect of lineage conversion is the efficient inhibition of the initial cell type. Overcoming the initial state is a well-described bottleneck in reprogramming to iPS cells^68,69^, and utilizing efficient initial lineage repression would require fewer cues to install a new cell identity. For example, miRNA-induced neuronal reprogramming of fibroblasts primarily acts via repression of the REST complex and non-neuronal factors, highlighting the importance of repression of the initial cell state^70^. Even though Ascl1 acts primarily as a transcriptional activator^6^, we show that Ascl1 rapidly downregulates the PPN. *Pou5f1* (Oct4) and *Nanog* are lost within 24 h upon Ascl1 expression, and *Sox2* is largely downregulated after 48 h. Similarly, MEF gene regulatory network is rapidly disassembled upon Ascl1 expression^7,71^. However, the Ascl1-induced pluripotency repression mechanism is yet to be addressed.

We conducted comparative forward genetic screens to systematically elucidate the genetic dependencies of both Ascl1 and Ngn2-induced transitions. Interestingly, Ascl1 transition was more dependent on the factors modulating the PPN and cell cycle. We selected Tcf7l1, a well-described PPN repressor, as a representative of this gene group. Surprisingly, the PPN was still downregulated even after *Tcf7l1* knockout. However, we noticed that Ascl1 fails to induce Cdkn1c in the absence of Tcf7l1. This suggests that exit from the cell cycle might be a prerequisite for Ascl1-driven neuronal conversion. Indeed, co-expression of Cdkn1c with Ascl1 triggered cell cycle exit and rescued the loss of Tcf7l1. Taken together, the ability of Ascl1 to rapidly downregulate the initial cell state and effectively induce cell cycle arrest can be an inherent part of its strong transdifferentiation potential across large developmental distances^27,72^. In such a case, efficient downregulation of the initial network would also allow easier installment of the new cellular state even from the weaker GRN induced by Ascl1. However, multiple signals induced by Ascl1 in the absence of initial state GRN would also allow permissive conditions for installing alternative cell fates, e.g., iT in ESC and myocyte in MEF.

In contrast, Ngn2 induces a stronger and more interconnected and self-sustaining network than Ascl1 in ESC. Indeed, Ngn2 gives rise to iN cells faster and more efficiently (Supplementary Fig. 1a). Similarly, Ngn2 binds to and induces more targets than Ascl1 also in human fibroblasts, including proneural genes like *NEUROD1* and *NEUROD4*^73,74^. Yet, only Ascl1 alone is sufficient to convert MEFs to functional neurons, while Ngn2 requires the addition of small molecules to facilitate genome accessibility^74,75^ or co-expression with additional transcriptional activators such as Brn3a^76,77^. This discrepancy could, at least in part, be explained by weaker repression of the initial state by Ngn2. For example, co-expression of transcriptional inhibitors, such as Myt1l, together with Ngn2 leads to successful MEF to iN conversion^62,76^. However, despite strong proneural GRN upregulation, Ngn2 induction leads to slower and incomplete repression of PPN after induction in ESC. This is indicative by the formation of trophoblast or primitive endoderm cells forming after and acute knockout of *Nanog* or Oct4, respectively, driven by the residual PPN^59–61^. Furthermore, Ngn2 retains *Sox2* expression as part of the NSC GRN, which acts as a proliferative intermediate. Thus, in ESCs, Ngn2 appears to overlay the PPN with neuron-specific genes and thereby generate a feed-forward loop to drive neuron formation with the appearance of NSC.

These observations indicate that Ascl1 and Ngn2 utilize different mechanisms to transition cells between identical states, namely from mESC to the same iN subtype. Specifically, Ascl1 dismantles one fate followed by the installment of another, while Ngn2 overlays the cell identities toward a new cell fate in a development-like process.

## Methods

The experiments in this study are in compliance with relevant guidelines and ethical regulations.

### Cell culture

Mouse ESCs (mESCs) were maintained in ESCM medium: DMEM (Sigma, D1152) supplemented with 15% Fetal bovine serum (FBS, Gibco, 10270106), 100 U/ml Penicillin, 100 μg/ml Streptomycin (Sigma, P0781), 1x Non-essential amino acids (Sigma, M7145), 4 mM L-Glutamine (Sigma, G7513), 1 mM Na-Pyruvate (Sigma, S8636), 0.1 mM β-mercaptoethanol (Merck, 805740), 50 μg/ml ascorbic acid, 1000 U/ml LIF (ESGRO Millipore). Cells were trypsinized and replated every second day on gelatin-coated plates with the irradiated DR4 mouse embryonic fibroblasts (MEF) feeders, isolated from the E13.5 stage embryo of the DR4 mouse strain (RRID:I MSR_JAX:003208), plated a day before.

For iN induction, mESC were trypsinized, and 10^8^ cells per well were plated on Matrigel (1 h 37 °C, 300 µL of 1000x diluted Matrigel (Szabo Scandic, BDL356231)) in a 24 well in the evening. The next day cells were washed twice with PBS and induced with N2B27 medium: DMEM/F12 (Gibco, 10565018), Neurobasal (Gibco, 21103049), N2 supplement (Gibco, 17502048), B27 supplement (Gibco, 17504044), 100 U/ml of Penicillin, 100 μg/ml Streptomycin (Sigma, P0781), 1x Non-essential amino acids (Sigma, M7145), 4 mM of L-Glutamine (Sigma, G7513), 1 mM of Na-Pyruvate (Sigma, S8636), 20 μg/mL Insulin (Roche, 15898200), 1 µg/ml doxycycline (dox). Time of induction was considered as day 0. From Day 2 onward, half of the medium was exchanged daily. For the induction of the iNSC state, cells were grown in the presence of 20 ng/ml FGF2 (PeproTech, 450-33) and 20 ng/ml EGF (PeproTech, 315-09). To enrich for non-dividing cells, 4 μM cytosine β-D-arabinofuranoside (AraC, Sigma-Aldrich) was added from day 4 onward. Cells were dissociated with trypsin and quantified using FACS BD LSR Fortessa (BD Biosciences). Subsequent gating of the data was done using Flowjo (BD Biosciences, v10.8.1).

### Cell lines

Doxycycline-inducible TetO-Ascl1 and TetO-Ngn2 mouse ESC lines were established as described previously^7^. Cells were then infected with a lentiviral construct containing constitutive Cas9. and single cell-derived clones were tested for stable Cas9 expression and robust ESC to iN conversion. Plasmid containing V5-P2A-Venus-T2A-PuroTK-LoxP-Ef1a-Neo-LoxP tag flanked with 750 bp homology arms adjacent to *Mapt* terminus was co-electroporated with a CRISPR-Cas9 sgRNA targeting *Mapt* gene C-terminus (Supplementary Table 1) using Mouse Embryonic Stem Cell Nucleofector™ kit (Lonza) and selected with 0.5 mg/ml G418 (Gibco). Neomycin resistance was excised via transfection of the plasmid containing Cre and individual clones were derived, genotyped, and tested for the reporter activity via FACS and immunostaining.

Individual clones with *Tcf7l1* knockouts were derived by infecting cells with the lentiviral vector containing sgRNA targeting *Tcf7l1* (Supplementary Table 1), mutagenizing for 7 days and subsequent picking of the single cell-derived clones. The correct genotype was confirmed by Sanger sequencing of the endogenous *Tcf7l1* locus and western blot.

N-terminus Tcf7l1 tagging with AID degron was done by co-electroporating plasmid containing mCherry-V5-AID flanked by 1kbp homology arms and a plasmid with sgRNA targeting *Tcf7l1* start codon. Successful homozygous integration in single cell-derived clones was confirmed via genotyping and western blot. Cells were infected with lentiviral vector delivering pSFFV-osTIR-T2A-eBFP2 and single cell-derived clones were tested for degradation of Tcf7l1, retained BFP expression during differentiation and showed no hypomorphic effects on iN conversion without induction of degradation.

An alternative cell line for validations of the formation of alternative lineages and ChIP-seq experiments was derived by electroporating E14 mESC (RRID:CVCL_C320) with piggyBac vector containing CAG-rtTA-IRES-Hygro (Addgene plasmid number #102423) and a plasmid with piggyBac transposase. Cells were then infected with a lentiviral construct containing Tet-O-FLAG-Ascl1-T2A-Puro or Tet-O-FLAG-Ngn2-T2A-Puro (modified from Addgene plasmid #52047) and single cell-derived clones were subsequently tested for the resistance to puro after dox addition.

To confirm the *Tcf7l1* knockout phenotype, cells were derived in triplicates in the same way described in the ChIP experiment, but without clonal expansion. Cells were then infected with an U6-sgTcf7l1-PGK-Cas9-P2A-Blast lentiviral vector, selected with 10 μg/ml blasticidin (Invivogen) for 4 days, and mutagenized for a week before assessing the phenotype.

For Cdkn1c co-expression with Ascl1, cells were infected with Tet-O-Cdkn1c-T2A-Puro (modified from Addgene plasmid #52047) vector. After 3 days, cells were induced as described above. From day 1, Cdkn1c expression was selected with 1 µg/ml Puromycin (Invivogen).

For PPN perturbation experiments, sgRNAs against *Nanog*, *Pou5f1*, *Sox2* were cloned into spCas9-P2A-Puro vector. One day post-infection, cells were selected with 1 µg/ml Puromycin (Invivogen) for a day. A kill control without Puromycin resistance cassette was used to ensure complete selection. Cells were then split and induced with Dox, as described above.

### SgRNA cloning and virus preparation

Individual sgRNAs (Supplementary Table 1) were cloned into the lentiviral plasmid containing spCas9-P2A-Blast using the single guide RNA Gecko cloning protocol (https://www.addgene.org/crispr/zhang). Lentiviral vectors were transfected using Polyethylenimine (PEI) into Lenti-X (Clontech, 632180) for virus production according to the supplier’s recommendations. Virus containing supernatant was filtered through a 0.45 μm PES filter (VWR). For infection, 10^8^ mESC were plated per gelatin-coated well in a 24-well plate. After recovery for at least 4 h, cells were infected with virus diluted 2x in fresh ESCM. The next day medium was exchanged and irradiated DR4 MEF feeders were plated on top of ESC. Successful integrations were selected with 10 μg/ml blasticidin for 4 days.

### Immunofluorescence

Cells were grown and differentiated on the Matrigel-coated glass coverslips. Cultured cells were then gently washed with PBS, fixed for 15 min at room temperature with 4% paraformaldehyde (PFA) in PBS. Cells were washed twice with PBS and subsequently permeabilized and blocked with blocking solution (5% FBS, 0.1% Triton™ X-100 in PBS) for 1 h at room temperature. Then coverslips were incubated with primary antibody overnight at 4 °C. Used primary antibodies were mouse anti-TUBB3 (Sigma, T8660, 1:500), rabbit anti-TUBB3 (Biolegend/Covance, PRB-435P, 1:500), rabbit anti-MAP2 (Abcam, ab32454, 1:500), rat anti-SOX2 (Invitrogen (eBioscience), 14-9811-80, 1:500), rabbit anti-OCT4 (Abcam, ab19857, 1:500), rabbit anti-NANOG (Abcam, ab80892, 1:500), rabbit anti-PAX6 (Covance, PRB-278P, 1:200), rat anti-KRT8/TROMA-I (DSHB, AB 531826, 1:200), rabbit anti-CDX2 (Abcam, ab76541, 1:400), mouse anti-Nestin (Merk, MAB353, 1:200), rat anti-GATA4 (Invitrogen, 14998082, 1:500), rabbit anti-CDKN1C (Abcam, ab75974, 1:250), rat anti-Mki67 (Invitrogen (eBioscience), 14-5698-82, 1:200), mouse anti-ASCL1 (Invitrogen (eBioscience), 14-5794-82, 1:200), rabbit anti-TPBPA (Abcam, ab104401, 1:200). Coverslips were washed three times with PBS 5 min at room temperature while gently rocking, and then incubated with secondary antibody in blocking solution for 1 h at room temperature. DAPI (0.5 μg/µl) was added together with a secondary antibody. Secondary antibodies used: goat anti-Mouse-488 (Invitrogen, A11029, 1:1000), goat anti-Rabbit-488 (Invitrogen, A11034, 1:1000), goat anti-Rat-488 (Invitrogen, A11006, 1:1000), goat anti-Mouse-568 (Invitrogen, A11031, 1:1000), goat anti-Rabbit-568 (Invitrogen, A11036, 1:1000), goat anti-Mouse-647 (Invitrogen, A21247, 1:1000), goat anti-Rabbit-647 (Invitrogen, A21236, 1:1000), goat anti-Rat-647 (Invitrogen, A21245, 1:1000). Coverslips were then washed as before and mounted on glass using Prolong™ Glass Antifade Mountant (Invitrogen, P36984).

For flow cytometry experiments with intracellular immunostaining of cells (FACS-IF), cells were grown as described above, dissociated with trypsin, washed with PBS and fixed for 15 min at room temperature with 4% PFA in PBS. Cells were then washed with PBS and 50–100 μl of cell suspension were pelleted at 400×*g* for 5 min, permeabilized and blocked with 100 μl blocking solution for 1 h at room temperature. Cells were then centrifuged at 400×*g* for 5 min and washed twice with 200 μl PBS. Cells were incubated with primary antibody (same as for microscopy samples) in the blocking solution for 1 h at room temperature, washed twice with 200 μl PBS with 400×*g* 5 min centrifugation in between, and incubated with secondary antibody (same as for microscopy samples) and DAPI (0.5 μg/µl) in the blocking solution for 1 h at room temperature. Cells were washed twice with 200 μl PBS and resuspended in 100 μl PBS and quantified using FACS BD LSR Fortessa (BD Biosciences).

### RT-qPCR

Total RNA was extracted using the Qiagen RNeasy mini kit. Then, 1–2 μg of total RNA was reverse transcribed using the SuperScript™ III Reverse Transcriptase (Invitrogen). RT-qPCR was performed using GoTaq® qPCR MasterMix (Promega). RT-qPCR primer sequences used in this study can be found in Supplementary Table 3. Relative RNA levels were calculated from *C*_*t*_ values by the standard ∆∆*C*_*t*_ method and normalized to *Actin* mRNA levels.

### Screen

Retroviral CRISPR-UMI sgRNA library was produced by transfection of the PlatinumE cells (Cell Biolabs) as described before^44^. In the morning, 3 × 10^8^ ESC were plated on the gelatin-coated plates (10^7^ cells per plate) without feeders and infected with a 10% infection rate 1 h later. In the evening, feeders were seeded on top of the ESC. Successfully infected cells were selected with 0.5 mg/ml G418 (Gibco, 11811-031) for 5 days changing medium daily and splitting while maintaining 500x library coverage at all times. Nine days post-infection, 5 × 10^6^ cells per plate were plated on Matrigel-coated 15 cm plates in the morning and induced with N2B27+Dox medium in the evening with a total of 6 × 10^7^ cells per condition. Half of the medium was exchanged daily. From day 4 onward, iN were purified by the addition of 4 μM cytosine β-D-arabinofuranoside (AraC, Sigma-Aldrich) and 1 μg/ml puromycin until day 6 post-induction. For the iNSC samples, cells were trypsinized and expanded after day 3 post-induction for additional 4 days in N2B27+Dox medium containing 20 ng/ml FGF2 (PeproTech, 450-33) and 20 ng/ml EGF (PeproTech, 315-09). iT were enriched by plating cells at day 6 in ESCM after trypsinization onto gelatin-coated 15 cm plates for 20 min and then washing off unattached cells with PBS. ESC were kept in parallel in ESCM medium and feeders as described above. At the end of differentiation, cells were trypsinized, washed with PBS, pelleted by centrifugation at 300×*g* for 5 min and frozen. Lastly, gDNA isolation, PacI digestion (NEB), size selection, PCR amplification and NGS were carried out as previously published^44,78^.

### RNAseq

Cells were collected at 0 h, 24 h and 48 h post-induction as bulk samples, and on days 3, 4, and 6, cells were sorted into Venus-positive and Venus-negative cells using FACSAria III (BD bioscience). Cells were washed with PBS and processed using the Qiagen RNeasy mini kit. RNA was additionally treated with a TURBO DNA-free™ kit (Invitrogen) according to the manufacturer’s protocols. The integrity of RNA was measured using Fragment Analyzer™ (Advanced Analytical). RNAseq-Libraries were prepared using QuantSeq 3’ mRNA-Seq Library Prep Kit (Lexogen GmbH). LUTHOR 3’-scRNAseq (Lexogen GmbH) libraries were prepared using the manufacturer’s protocol after sorting cells into 5 μl lysis agent. Concentrations and distributions of the libraries were checked with the Fragment Analyzer™ using HS NGS Fragment Kit (Agilent; DNF-474-0500). Libraries were then pooled and sequenced using Illumina Nextseq550 in a single read 75 cycles run.

### ChIPseq

Two replicates of 25 × 10^6^ E14 (CAG-rtTA-Hygro, LV-TetOL-Flag-Ascl1/Ngn2-T2A-Puro) cells were collected 24 h after induction, washed with PBS and fixed with 1% freshly prepared formaldehyde (FA) solution in PBS for 7 min at room temperature. FA was then quenched with 0.125 M glycine. Nuclei were extracted by cell lysis in 50 mM HEPES-KOH pH 8.0, 140 mM NaCl, 1 mM EDTA, 10 % glycerol, 0.5% NP40, 0.25 % Triton X-100 for 10 min at 4 °C, followed by 5 min on ice of 10 mM Tris-HCl pH 8.0, 1 mM EDTA, 0.5 mM EGTA, 200 mM NaCl. Nuclei were then washed and resuspended in 10 mM Tris-HCl pH 8.0, 0.1 % SDS, 1 mM EDTA with 1x Complete mini protease inhibitors (Roche), and chromatin was sheared using Covaris E220 High Performance Focused Ultrasonicator (Duty factor 5.0, PIP 140.0, 200 cycles per burst at 4 °C). Fragment sizes of 250–800 bp were confirmed via agarose gel electrophoresis. 1% of fragmented chromatin was kept as an input sample. For each replicate, a duplicate of 50 μg of chromatin was used for overnight 4 °C incubation with 5 μg of anti-FLAG (Sigma, F1804) in IP Buffer (50 mM HEPES-KOH pH 7.5, 140 mM NaCl, 1 mM EDTA, 1% Triton X-100, 0.1% DOC, 0.1% SDS) with BSA blocked Protein G coupled Dynabeads (Thermo Fisher Scientific; Blocking was done for 3 h at 4 °C). Beads were then washed eight times with IP buffer and once with TE, 50 mM NaCl. The DNA was eluted twice with 150 μl of 1% SDS, 0.1 M NaHCO_3_ for 20 min at 65 °C. The eluate was then treated with RNase A and Proteinase K and reverse crosslinked overnight at 65 °C. DNA was purified using phenol/chloroform extraction and isopropanol precipitation. ChIP libraries were prepared using the NEBNext Ultra-II kit (New England Biolabs) following the manufacturer’s protocol. The quality of the libraries was assessed with the Fragment Analyzer™ using HS NGS Fragment Kit, and libraries were sequenced using Illumina HiSeq V4 in a single read 50 bp sequencing run.

### Data analysis

#### RNAseq

RNA-seq reads were trimmed using BBDuk v38.06 (ref=polyA.fa.gz,truseq.fa.gz k = 13 ktrim=r useshortkmers=t mink=5 qtrim=r trimq=10 minlength=20) and reads mapping to abundant sequences included in the iGenomes Ensembl GRCm38 bundle (mouse rDNA, mouse mitochondrial chromosome, phiX174 genome, adapter) were removed using bowtie2 v2.3.4.1 alignment. The remaining reads were analyzed using genome and gene annotation for the GRCm38/mm10 assembly obtained from *Mus musculus* Ensembl release 94. Reads were aligned to the genome using star v2.6.0c, and reads in genes were counted with featureCounts (subread v1.6.2) using strand-specific read counting for QuantSeq experiments (-s 1). For differential expression analysis and variance stabilized transformation for PCA analysis, we used R package Deseq2^79^ (v.1.32.0). Significantly differentially expressed genes (DEG) were considered those genes with FDR < 0.05 and −1 <Log2(Fold change) <1. For the KEGG pathway enrichment analysis and identification of the alternative cell lineages using the PanglaoDB database^32^, we used Enrichr^80^. For the DEG network analysis and visualization, we used the STRING database and Cytoscape (v.3.8.0).

#### ChIPseq

ChIP-seq reads were trimmed using trim-galore v0.4.4 and thereafter aligned to the mm10 reference genome using bwa mem v0.7.17. Duplicated reads were removed using Picard MarkDuplicates (v2.23.4.). Complexity and overall data quality were assessed using phantompeakqualtools and deepTools plotFingerprint (v 3.5.0). To generate bigwig files from corresponding bam files, we used deepTools bamCoverage (v 3.5.0), with parameter -normalizeUsing RPKM. Peak calling from the sorted BAM files was done using MACS2 (v2.1.1). Peaks in blacklist regions were identified using bedtools intersect (v2.27.1) and mm10.blacklist.bed.gz v1. To analyze overlapping and unique peaks between Ascl1 and Ngn2, as well as plotting peak heatmaps, we used the R package Diffbind^81^ (v3.2.7) with minOverlap = 4 to collapse replicates after making a consensus peaksets. For determining binding motifs, we used MEME-ChIP (v5.1.1) with -meme-minw 6 -meme-maxw 15 parameters. For plotting the binding profiles, we combined the reads from the replicates using UCSC bigWigMerge and used R package karyoploteR (v1.18.0).

#### CRISPR-Cas9 screen analysis

Analysis of CRISPR-Cas9 screens was done according to previously published protocol^44,78^. In short, reads were trimmed to 20 nt sgRNA sequence using fastx-toolkit (v0.0.14) and mapped to reference sequences using bowtie (v1.1.2). Experimental indices and mapped reads were collapsed into count tables using in-house Python scripts. Gene enrichment was determined using MAGeCK^82^ (v0.5.4). Visualization was done using the R package ggplot2 (v3.3.6).

#### Statistics and reproducibility

Experiments were independently performed at least three times (unless otherwise stated). Figure 3g screen validation experiment, a replicate was excluded for Setdlb due to an inefficient number of cells, thus resulting in poor sample quantification. No key conclusions were drawn based on this sample. Results are reported as mean ± SD. For comparing between two groups, a two-tailed unpaired Student’s t-test was performed. One-way ANOVA followed by Dunnett’s multiple comparison test was applied for comparisons of multiple groups. Statistical tests were performed using R. Only representative micrographs are shown from at least two independent replicates, as similar results were obtained between replicates. In Supplementary Figs. 4, 12c–d, micrographs of all the replicates are shown.

### Reporting summary

Further information on research design is available in the Nature Portfolio Reporting Summary linked to this article.

### Supplementary information

Supplementary Information

Reporting Summary

### Source data

Source data

## Data Availability

Raw NGS data produced in this study have been deposited in Gene Expression Omnibus (GEO) database under super series accession number GSE206872: ChIP-seq (GSE206869), RNA-seq (GSE206870, GSE208199), CRISPR-Cas9 screen (GSE206871). Single-cell RNA-seq data for reanalysis can be obtained from GSE125620^6^. Source data are provided with this paper.

## Code availability

Only standard bioinformatic code was used for the data analysis and is available on request.
