# Supplementary material for: Ascl1 and Ngn2 convert mouse embryonic stem cells to neurons via functionally distinct paths
Source: Nat Commun. 2023 Sep 2;14:5341. doi: 10.1038/s41467-023-40803-y (PMC10475046; doi:10.1038/s41467-023-40803-y)
Supplement: Supplementary file 1 — Supplementary Information [file 41467_2023_40803_MOESM1_ESM.pdf]

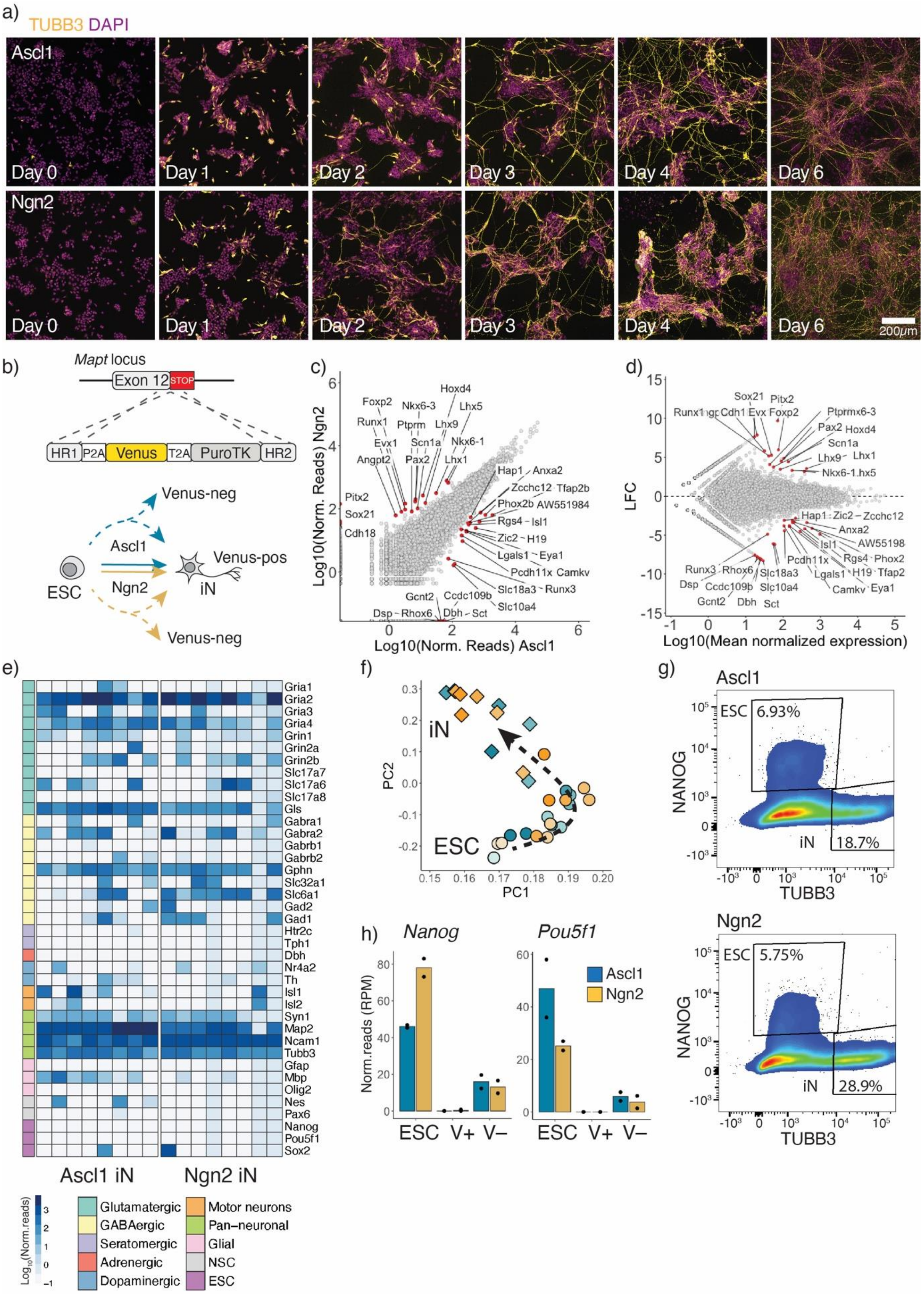
a)
TUBB3 DAPI
Ascl1
Ngn2
Day 0
Day 1
Day 2
Day 3
Day 4
Day 6
200μm
b)
Mapt locus
Exon 12
STOP
HR1
P2A
Venus
T2A
PuroTK
HR2
Venus-neg
Ascl1
Venus-pos
ESC
Ngn2
iN
Venus-neg
c)
Log10(Norm. Reads) Ngn2
Log10(Norm. Reads) Ascl1
d)
LFC
Log10(Mean normalized expression)
e)
Gria1
Gria2
Gria3
Gria4
Grin1
Grin2a
Grin2b
Slc17a7
Slc17a6
Slc17a8
Gls
Gabra1
Gabra2
Gabrb1
Gabrb2
Gphn
Slc32a1
Slc6a1
Gad2
Gad1
Htr2c
Tph1
Dbh
Nr4a2
Th
Isl1
Isl2
Syn1
Map2
Ncam1
Tubb3
Gfap
Mbp
Olig2
Nes
Pax6
Nanog
Pou5f1
Sox2
Ascl1 iN
Ngn2 iN
Glutamatergic
GABAergic
Seratomergic
Adrenergic
Dopaminergic
Motor neurons
Pan−neuronal
Glial
NSC
ESC
f)
PC2
PC1
iN
ESC
g)
Ascl1
ESC
6.93%
NANOG
iN
18.7%
TUBB3
Ngn2
ESC
5.75%
NANOG
iN
28.9%
TUBB3
h)
Nanog
Pou5f1
Norm.reads (RPM)
Ascl1
Ngn2
ESC
V+
V–

Supplementary Figure 1. Characterization of induced neurons at day 6

a, Immunostainings showing forward differentiation of ESC over time after overexpression of Ascl1 or Ngn2. **b**, Scheme of C-terminally tag of Mapt-Venus and a visual representation of ESC differentiating towards iN after Ascl1 or Ngn2 induction. Cells may take a common path (solid line) or different paths (dashed line) between similar cell states, generating to Mapt-Venus positive iN population, while failure to reprogram or generating alternative cell states will result in Mapt-Venus negative population. **c,d**, Scatter plot (**c**) and MA plot (**d**) comparing gene expression at Day 6 between Ascl1 and Ngn2 Venus positive cells (Fig. 1b). Differentially expressed genes are indicated in red (FDR < 0.05). **e**, Heatmap showing neuronal subtype specific markes expressed in individual neurons using LUTHOR 3' ultra-high sensitivity scRNAseq. **f**, Combined principal component analysis of time resolved bulk RNAseq (Fig. 1 c,d). **g**, FACS-IF plots showing intracellular immunostaining of the cells for pan-neuronal marker TUBB3 and core pluripotency marker NANOG at day 6. **h**, Expression of pluripotency genes at day 6 in the mRNA dataset in the Fig. 1a. Bar plot shows mean of n=2 independent biological replicates. V – Mapt-Venus expression.

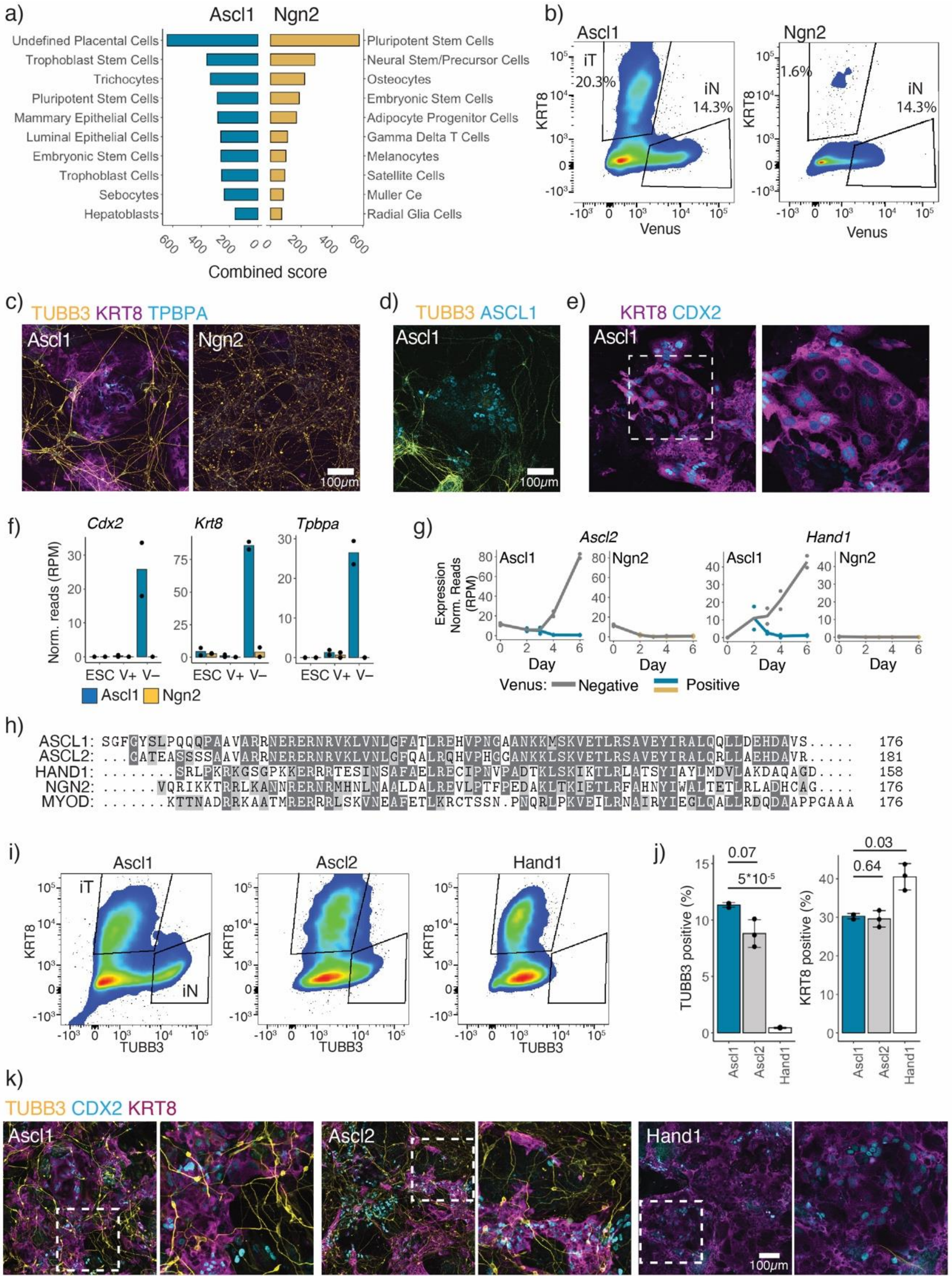
a)
Ascl1
Ngn2
Undefined Placental Cells
Trophoblast Stem Cells
Trichocytes
Pluripotent Stem Cells
Mammary Epithelial Cells
Luminal Epithelial Cells
Embryonic Stem Cells
Trophoblast Cells
Sebocytes
Hepatoblasts
Pluripotent Stem Cells
Neural Stem/Precursor Cells
Osteocytes
Embryonic Stem Cells
Adipocyte Progenitor Cells
Gamma Delta T Cells
Melanocytes
Satellite Cells
Muller Ce
Radial Glia Cells
Combined score
b)
Ascl1
Ngn2
iT
20.3%
iN
14.3%
1.6%
iN
14.3%
KRT8
Venus
c)
TUBB3 KRT8 TPBPA
Ascl1
Ngn2
100μm
d)
TUBB3 ASCL1
Ascl1
100μm
e)
KRT8 CDX2
Ascl1
f)
Cdx2
Krt8
Tpbpa
Norm. reads (RPM)
ESC V+ V−
Ascl1
Ngn2
g)
Ascl2
Hand1
Ascl1
Ngn2
Expression Norm. Reads (RPM)
Day
Venus:
Negative
Positive
h)
ASCL1: SGFGYSLPQQQPAAVARRNERERNRVKLVNLGFATLREHVPNGAANKKMSKVETLRSAVEYIRALQQLLDEHDAVS..... 176
ASCL2: ...GATEASSSSAAVARRNERERNRVKLVNLGFQALRQHVPHGGANKKLSKVETLRSAVEYIRALQRLLAEHDAVR..... 181
HAND1: ........SRLPKRKGSGPKKERRRTESINSAFAELRECIPNVPADTKLSKIKTLRLATSYIAYLMDVLAKDAQAGD.... 158
NGN2: ......VQRIKKTRRLKANNRERNRMHNLNAALDALREVLPTFPEDAKLTKIETLRFAHNYIWALTETLRLADHCAG.... 176
MYOD: ........KTTNADRRKAATMRERRRLSKVNEAFETLKRCTSSN.PNQRLPKVEILRNAIRYIEGLQALLRDQDAAPPGAAA 176
i)
Ascl1
Ascl2
Hand1
iT
iN
KRT8
TUBB3
j)
0.07
5*10-5
0.03
0.64
TUBB3 positive (%)
KRT8 positive (%)
Ascl1
Ascl2
Hand1
k)
TUBB3 CDX2 KRT8
Ascl1
Ascl2
Hand1
100μm

Supplementary Figure 2. Identification of side population formed after Ascl1 overexpression

a, Cell type prediction using PanglaoDB from differentially upregulated genes at day 6 (Fig. 1e, f). Combined score is defined in Kuleshov et al, 2016. **b**, Cells immunostained for Mapt-Venus and KRT8, indicating neuronal and trophoblast populations, respectively, at day 6 post Ascl1 or Ngn2 induction. **c**, Cells immunostained for the trophoblast markers KRT8 and TPBPA and neuronal marker TUBB3 at day 6 post Ascl1 induction. Trophoblast markers were expressed after Ascl1 induction, and not Ngn2. **d**, Cells immunostained for the Ascl1 expression at day 6 post induction. **e**, Binucleated trophoblast like cells stained for immunostained for the trophoblast markers KRT8 and CDX2 at day 6 post induction of Ascl1. **f**, Expression of trophoblast marker genes at day 6 in the mRNA dataset in the Fig. 1a. Bar plot shows mean of n=2 biologically independent samples. V – Mapt-Venus expression. **g**, Time course (Fig. 1a) of the expression of bHLH transcription factors *Ascl2* and *Hand1* after induction of Ascl1 or Ngn2. Lines are drawn through the mean of n=2 biologically independent replicates. **h**, Comparison of the bHLH DNA binding domain amino acid sequences of the neural factors ASCL1, NGN2; trophoblast - ASCL2, HAND1; skeletal muscle – MYOD. The shades of letter highlights represent the similarities of the amino acids. **i**, Immunostaining for trophoblast marker KRT8 and neuronal marker TUBB3 at day 6 post induction of bHLH transcription factors Ascl1, Ascl2, Hand1. **j**, Quantification of iN and iT formation by cells immunostaining (**i**) for TUBB3 and KRT8, respectively, after 6 days of Ascl1, Ascl2 and Ngn2 induction. N = 3 biologically independent samples. Barplot indicates mean with ± SD. P values of the two-sided Welch two sample t test indicated above. **k**, Representative immunostained cells for trophoblast markers CDX2 and KRT8 and neuronal marker Tubb3 at day 6 post induction of pro-neural Ascl1, trophoblast lineage driving Ascl2 or Hand1 bHLH transcription factors. Neurons were induced after Ascl1 and Ascl2 expression, but not Hand1, while trophoblast cells were present upon induction of all three factors.

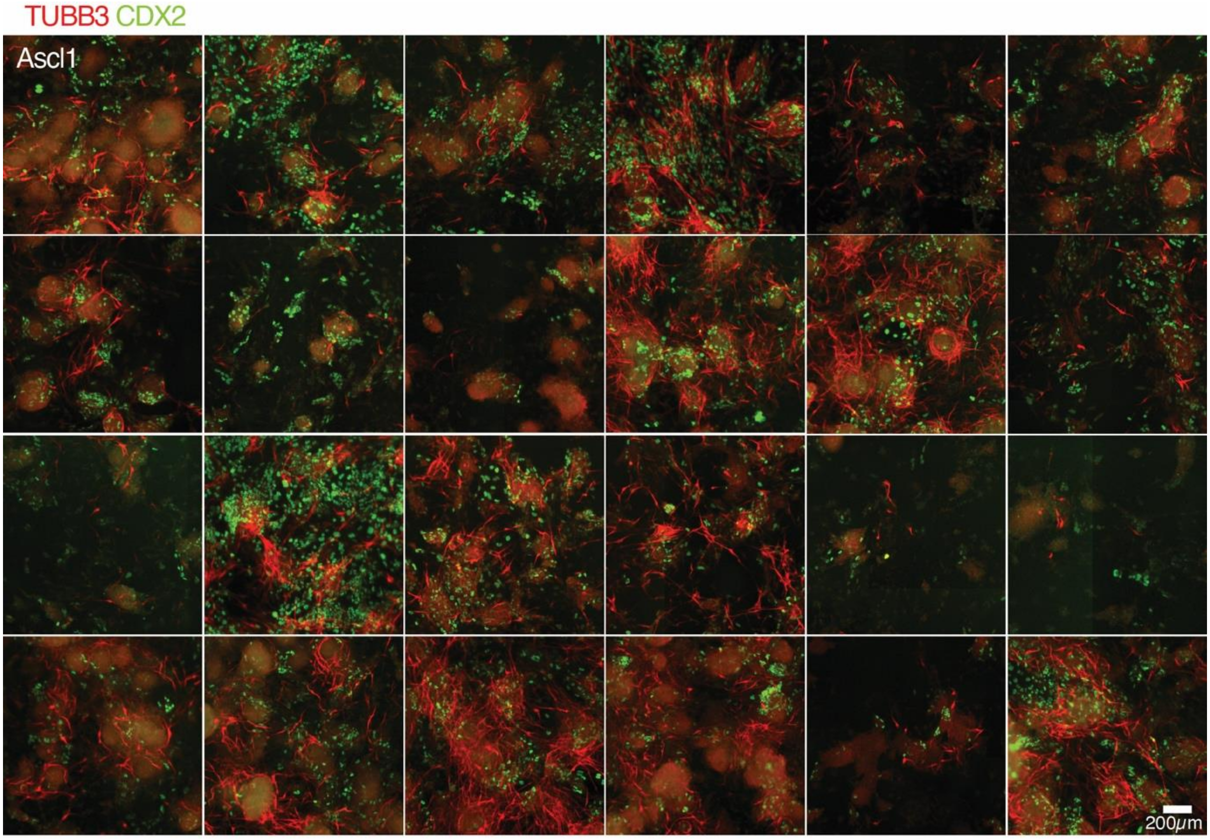

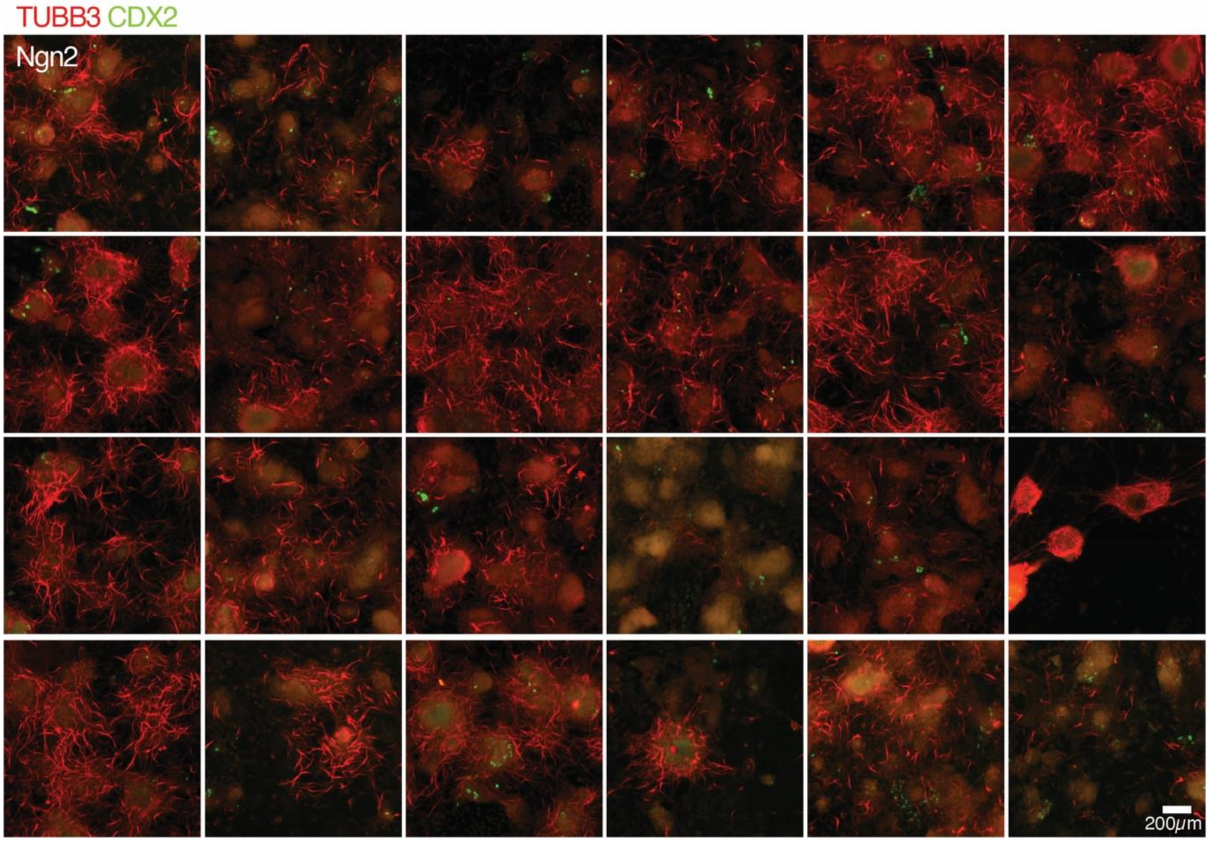

Supplementary Figure 3. Trophoblast formation in single cell derived Ascl1 or Ngn2 expressing clones
Expression of trophoblast CDX2 and neuronal TUBB3 markers in independently derived ESC clones with Ascl1 or Ngn2 overexpression cassette.

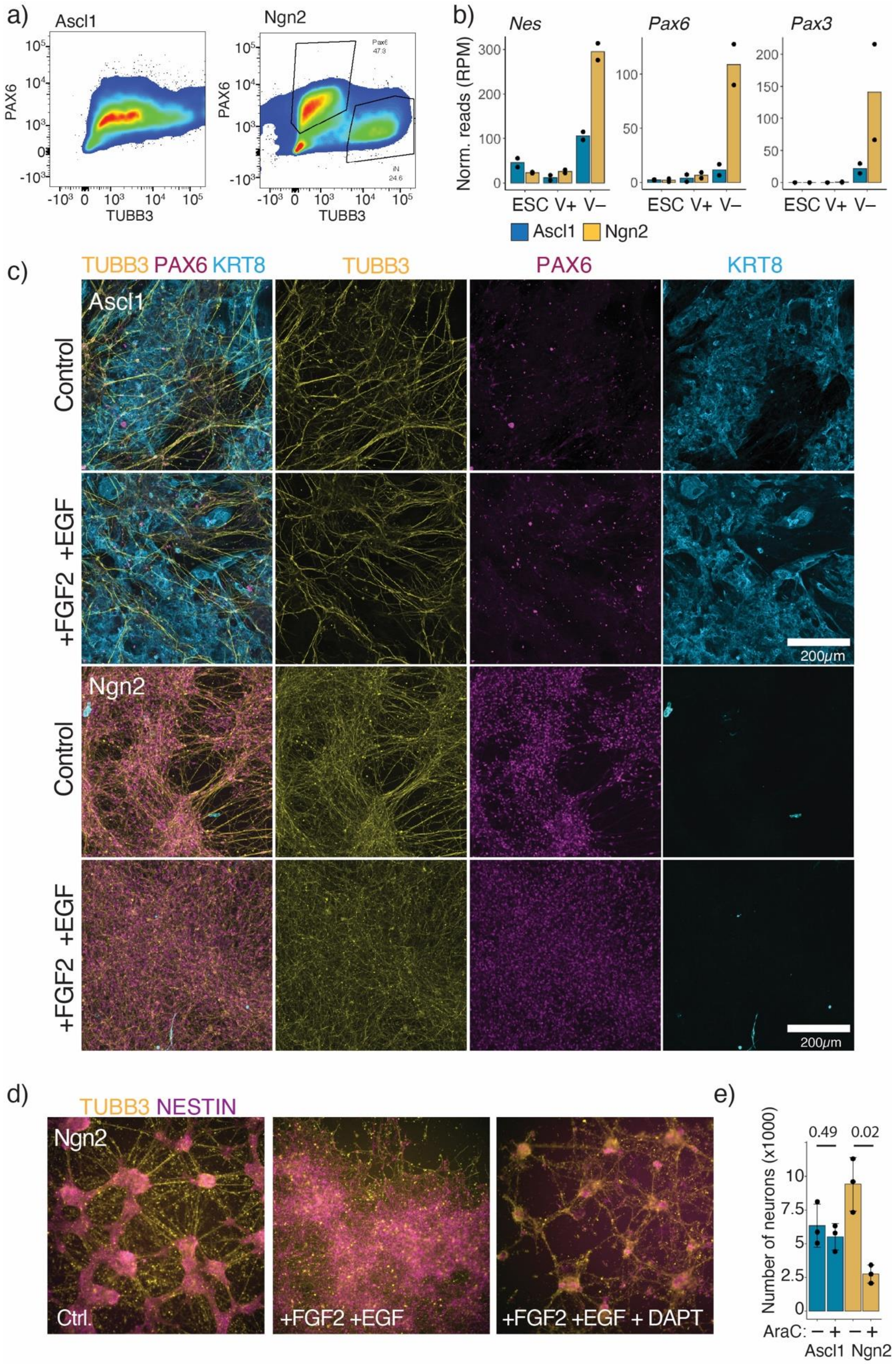

a)
Ascl1
Ngn2
PAX6
TUBB3
Pax6
47.3
iN
24.6
b)
Nes
Pax6
Pax3
Norm. reads (RPM)
ESC V+ V–
Ascl1
Ngn2
c)
TUBB3 PAX6 KRT8
TUBB3
PAX6
KRT8
Ascl1
Control
+FGF2 +EGF
Ngn2
200μm
d)
TUBB3 NESTIN
Ngn2
Ctrl.
+FGF2 +EGF
+FGF2 +EGF + DAPT
e)
Number of neurons (x1000)
0.49
0.02
AraC: – + – +
Ascl1 Ngn2

Supplementary Figure 4. Characterization of NSC-like population Ngn2 overexpression

a, Cells immunostained for TUBB3 and PAX6, indicating neuronal and NSC-like populations, respectively, after day 6 of Ascl1 or Ngn2 induction. **b**, Expression of NSC marker genes at day 6 in the mRNA dataset in the Fig. 1a. Bar plot shows mean of n=2 biologically independent samples . V – Mapt-Venus expression. **c**, Cells immunostained for neuronal marker TUBB3, trophoblast marker KRT8, NSC marker PAX6 at 6 day post induction of Ascl1 or Ngn2 in the presence of NSC inducing factors FGF2, EGF. **d**, Ngn2 induced cells immunostaining for neuronal marker TUBB3 and NSC marker NESTIN at day 6 post induction in the presence of NSC inducing factors FGF2, EGF, and Notch inhibitor DAPT. **e**, Number of neurons as measured by Mapt-Venus reporter in the presence or absence of AraC from day 4 post induction. N = 3 biologically independent samples. Bar plot shows mean with ± SD. P values of the two-sided Welch two sample t test indicated above.

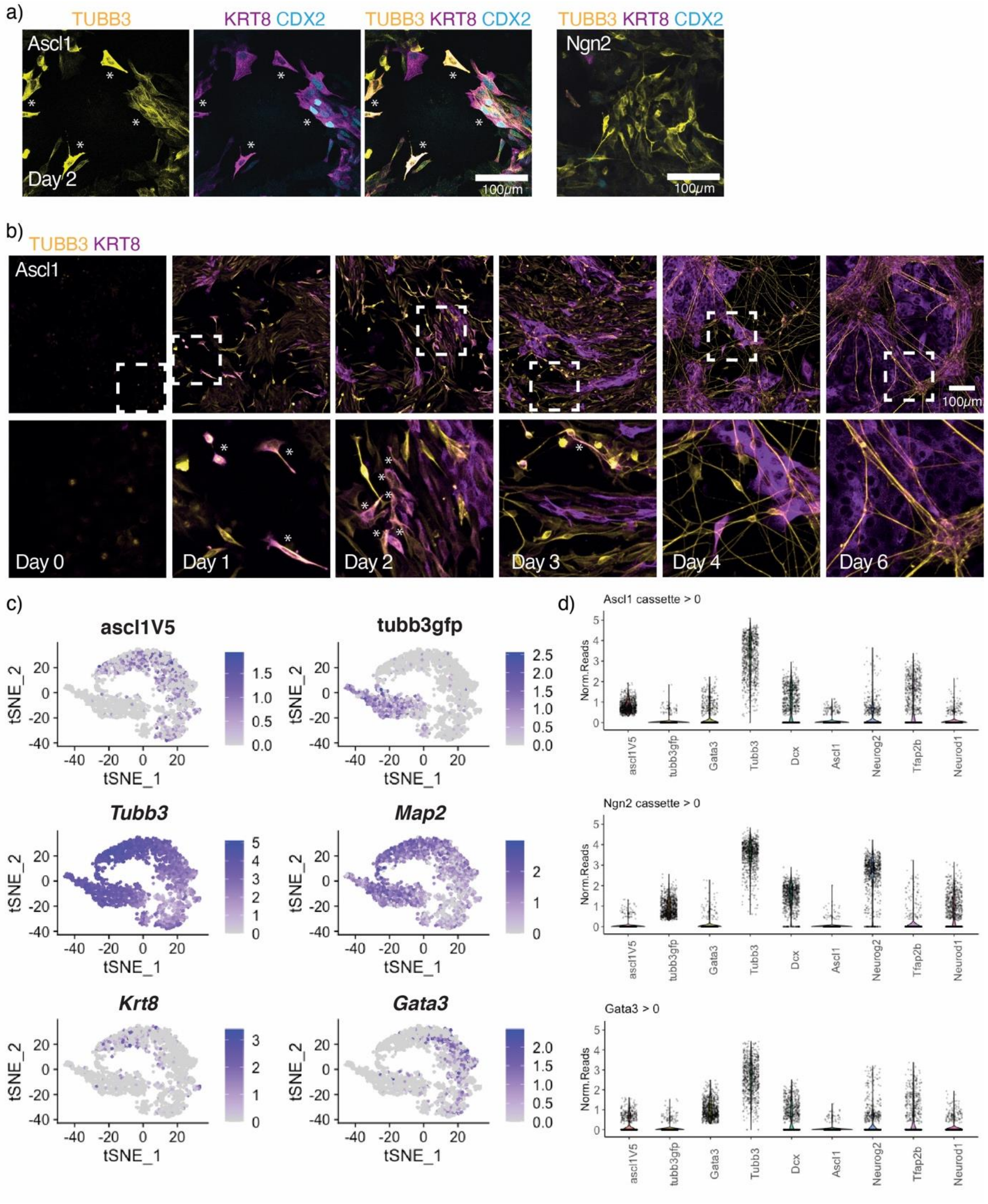

Supplementary Figure 5. iN and iT lineages coexist in early Ascl1 induced ESC to iN conversion

a, Cells immunostained for the neuronal TUBB3 and trophoblast markers CDX2, KRT8 at day 2 after Ascl1 induction. Asterisks indicate cells co-staining for both neuronal and trophoblast markers. **b**, Time resolved immunostainings of cells for neuronal TUBB3 and trophoblast marker KRT8. Asterisks indicate cells co-staining for both neuronal and trophoblast markers. **c**, scRNAseq data generated by Aydin et. al. 2019 showing trophoblast markers *Gata3*, *Krt8* expressed in Ascl1 (labeled as ascl1V5), but not Ngn2 (labeled tubb3gfp), induced cells. **d**, Cells filtered for expression by overexpression cassette (Ascl1 – ascl1V5, Ngn2 – tubb3gfp) or *Gata3*. Ascl1 induced cells co-express both neuronal *Tubb3*, *Dcx2* as well as trophoblast *Gata3* markers.

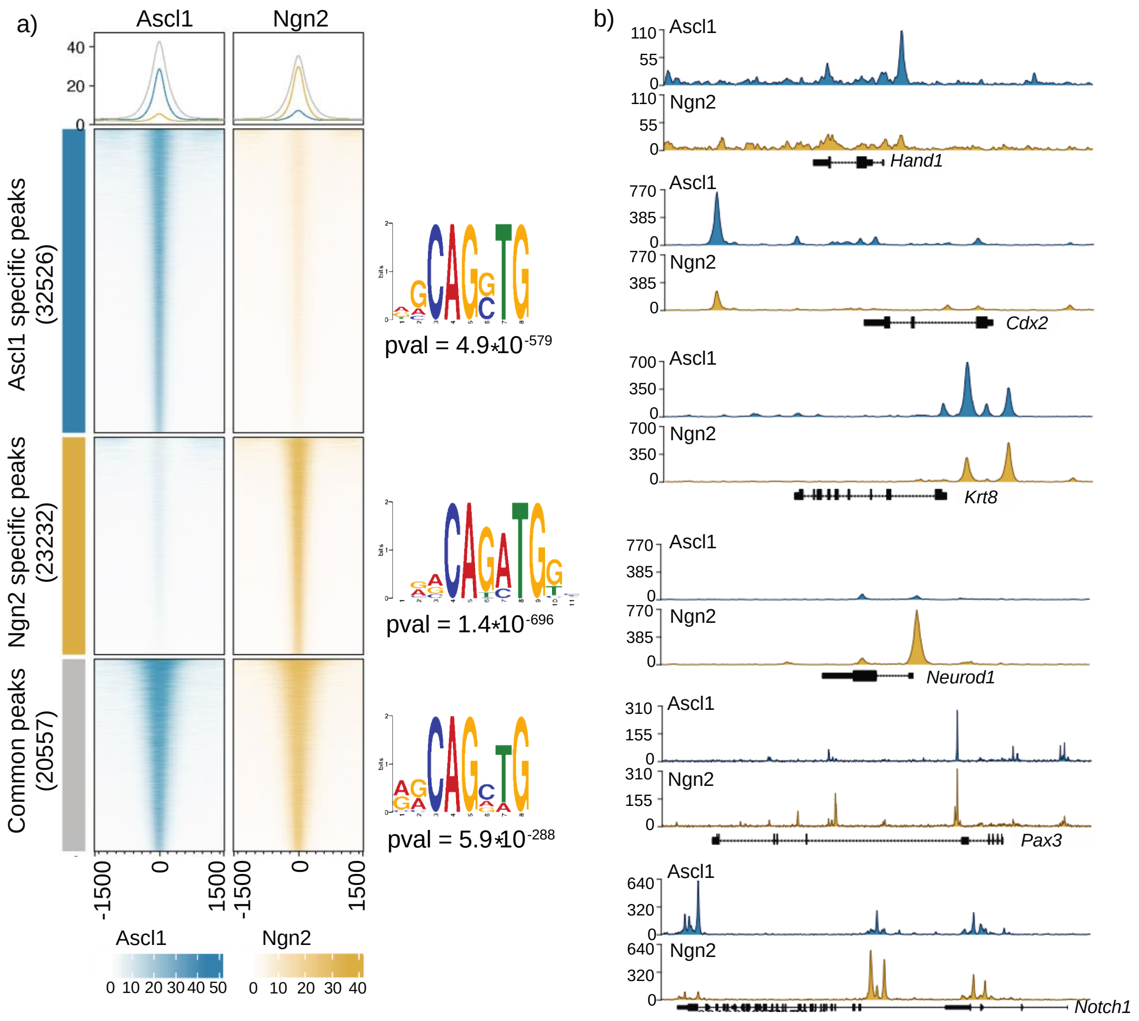

Supplementary Figure 6. Ascl1 and Ngn2 binding analysis

a, ChIPseq heatmap showing Flag-Ascl1 and Flag-Ngn2 binding 1 day after induction. Peaks are grouped based on binding by Ascl1, Ngn2 or both, with the top enriching motifs for the corresponding groups on the right. Peaks are plotted on the heatmap with a ± 1.5kb window around the peak center (n = 4). **b**, Examples of ChIP profiles showing binding of Ascl1 or Ngn2 to alternative lineage genes.

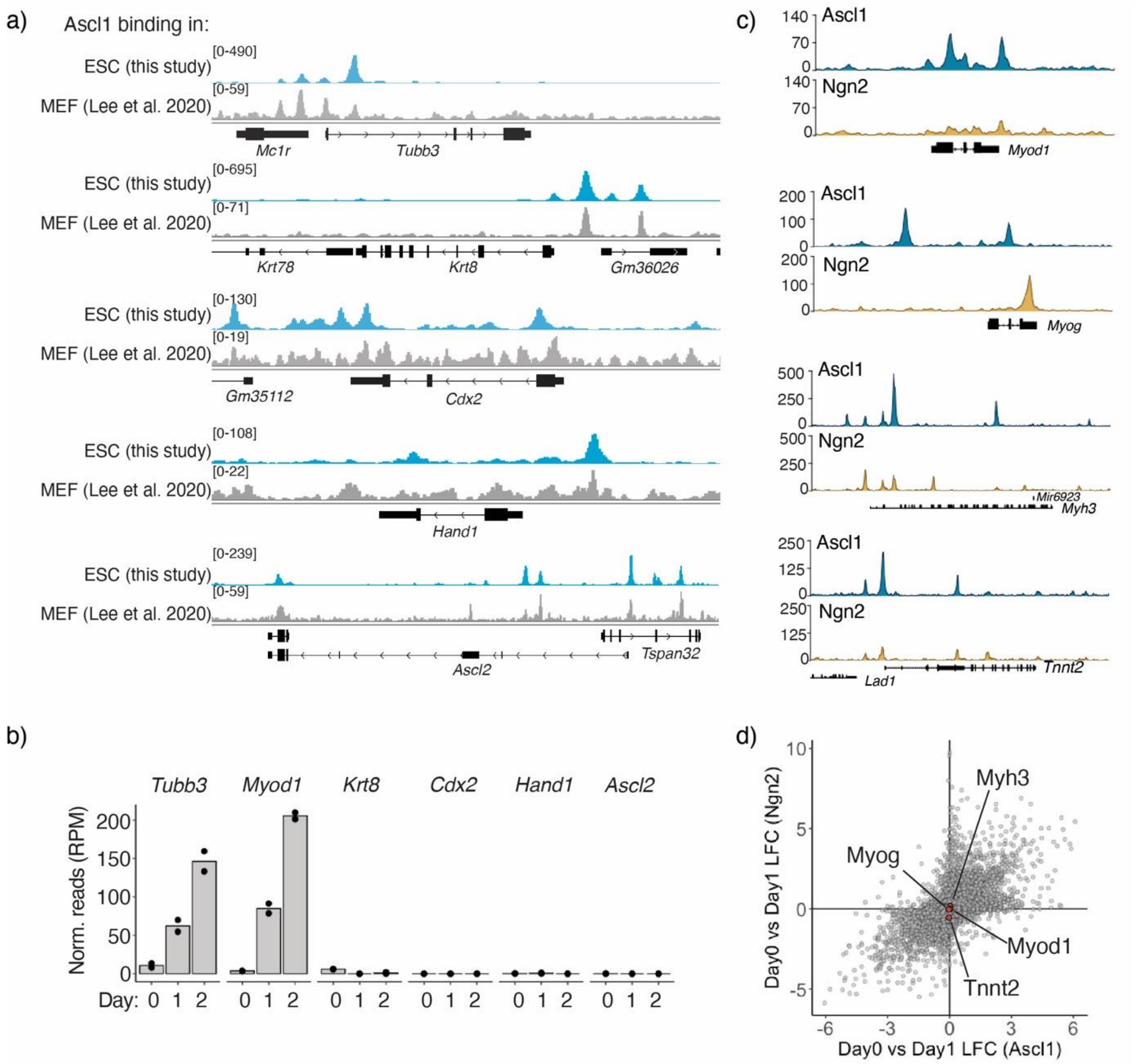

Supplementary Figure 7. Ascl1 binding of alternative lineage markers in ESC and MEF

a, Examples of ChIP profiles showing binding of Ascl1 to the trophoblast lineage markers in ESC and MEF. **b**, Bulk RNAseq expression profiles of the trophoblast lineage markers (**a**) 1 and 2 days post induction of Ascl1 in MEF. Bars show mean of n=2 independent biological replicates. **c**, Examples of ChIP profiles showing Ascl1 or Ngn2 binding of the muscle lineage markers in the ESC. **d**, Expression of muscle lineage markers (**c**) 1 day post induction of Ascl1 or Ngn2 (Fig. 1i).

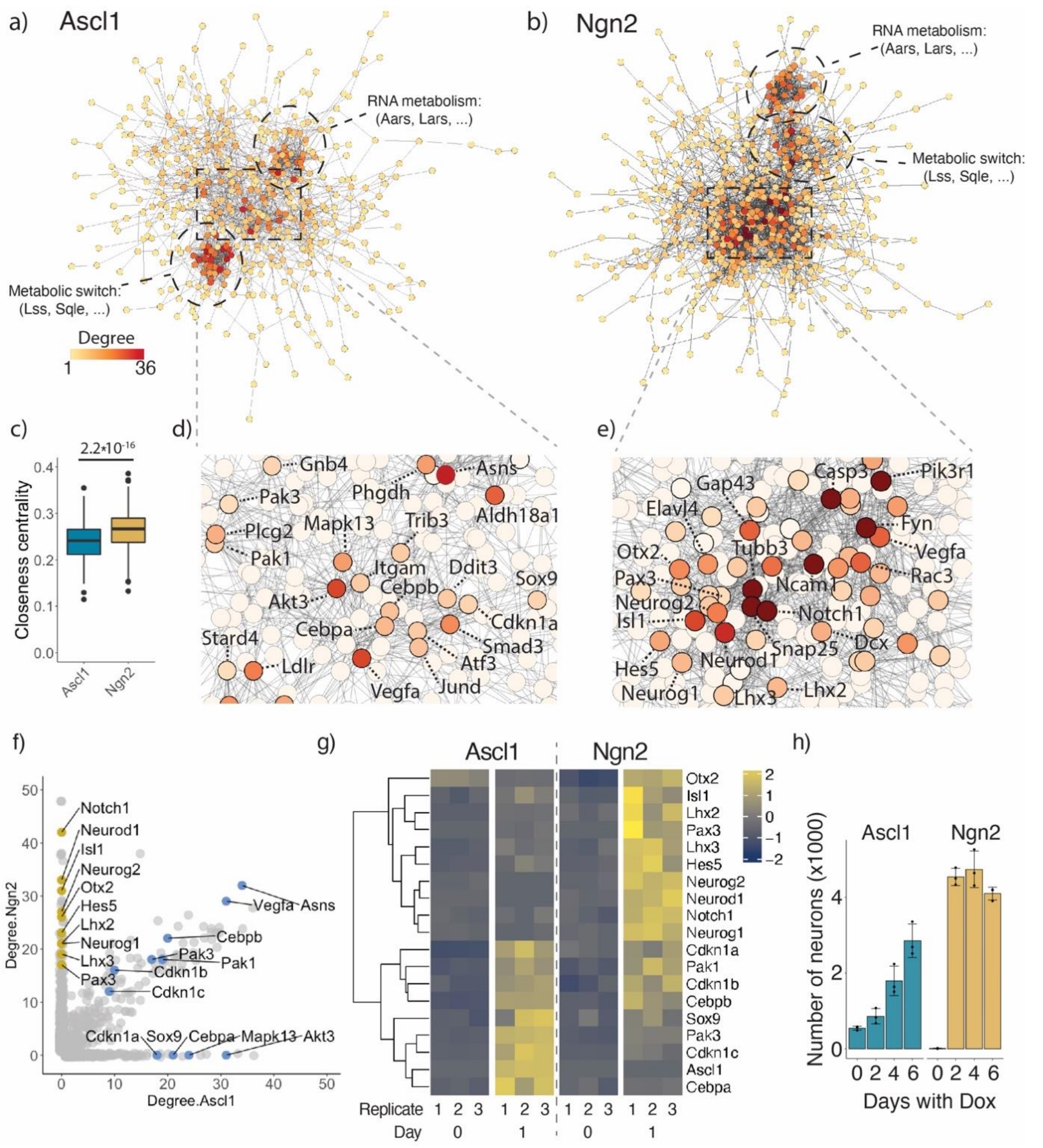

Supplementary Figure 8. Differences in transcriptional responses between Ascl1 or Ngn2 induction

a – b, STRING network reconstruction for Ascl1 (**a**) or Ngn2 (**b**) significantly upregulated genes at day 1 post induction (n = 3, LFC > 1, FDR < 0.05). Values indicate degree of connectivity of a particular gene. Oval dashed line indicates common gene groups between Ascl1 and Ngn2 related to metabolism. Dashed box indicates central nodes in (**d**) and (**e**). **c**, Closeness centrality measure of the (**a**) and (**b**) networks. Boxplots indicate 25th and 75th percentiles as bounds of the box with the median centre line, whiskers indicate minima/maxima of a 1.5x distance of the IQR from the 25th and 75th percentiles, dots indicate outliers outside minima/maxima range. P value of the two-sided Welch two sample t test indicated above. **d – e**, central nodes of the (**a**) and (**b**) networks. Nodes with highest degree of connectivity are labeled. **f**, Scatter plot comparing degree of connectivity between Ascl1 and Ngn2 expressed genes. Central nodes with highest degree of connectivity for Ascl1 are labeled in blue, and highly interconnected neuronal lineage driving genes for Ngn2 are labeled in yellow. **g**, Heatmap showing gene expression for neuronal lineage drivers and example highly interconnected central nodes in Ascl1 expression network (**e**). Color indicates row normalized vst transformed normalized expression of data from Fig. 1i. **h**, Number of neurons as measured by Mapt-Venus reporter after different days of induction of the cassette. Days indicate the period of Dox treatment. Bar plot shows mean of n = 3 biologically independent samples with ± SD.

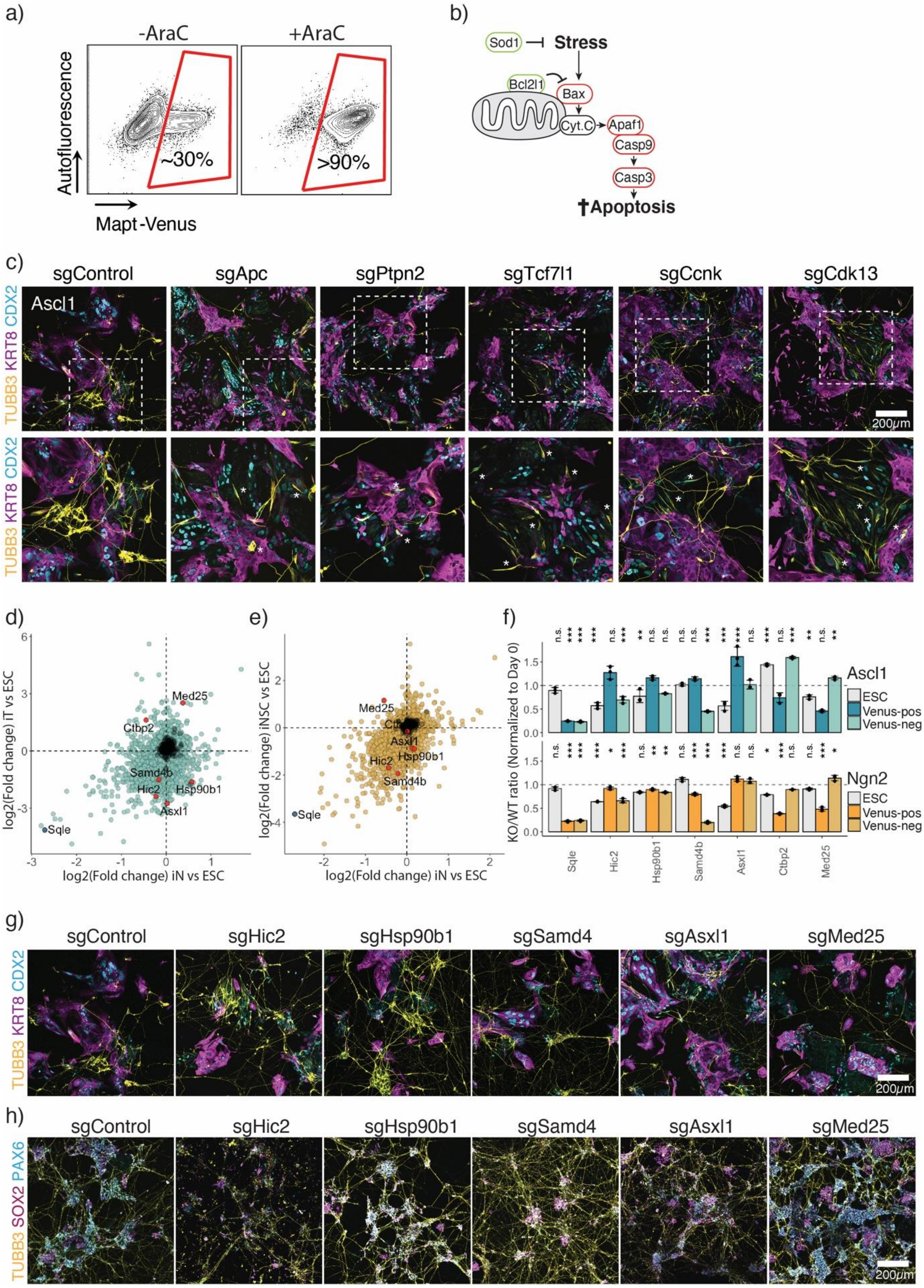
a)
-AraC
+AraC
Autofluorescence
Mapt-Venus
~30%
>90%
b)
Sod1
Stress
Bcl2l1
Bax
Cyt.C
Apaf1
Casp9
Casp3
†Apoptosis
c)
sgControl
sgApc
sgPtpn2
sgTcf7l1
sgCcnk
sgCdk13
Ascl1
TUBB3 KRT8 CDX2
200μm
d)
log2(Fold change) iT vs ESC
log2(Fold change) iN vs ESC
Med25
Ctbp2
Samd4b
Hic2
Hsp90b1
Asxl1
Sqle
e)
log2(Fold change) iNSC vs ESC
log2(Fold change) iN vs ESC
Med25
Ctbp2
Asxl1
Hsp90b1
Hic2
Samd4b
Sqle
f)
KO/WT ratio (Normalized to Day 0)
Ascl1
Ngn2
ESC
Venus-pos
Venus-neg
Sqle
Hic2
Hsp90b1
Samd4b
Asxl1
Ctbp2
Med25
g)
sgControl
sgHic2
sgHsp90b1
sgSamd4
sgAsxl1
sgMed25
TUBB3 KRT8 CDX2
200μm
h)
sgControl
sgHic2
sgHsp90b1
sgSamd4
sgAsxl1
sgMed25
TUBB3 SOX2 PAX6
200μm

Supplementary Figure 9. Additional CRISPR-Cas9 screen results

a, Purification of Mapt-Venus positive cells by AraC addition at day 4 post induction. Red gate indicating Mapt-Venus positive iN cells. **b**, A reduced scheme depicting apoptosis pathway. Colored proteins indicate hits in the screen: apoptosis promoting and enriching in the screen (red), and apoptosis preventing and depleting in the screen (green). **c**, Immunostaining of the knockout cells for neuronal marker TUBB3 and alternative lineage markers KRT8 / CDX2 for Ascl1 induced iT (Fig. 2h), with TUBB3 positive cells lacking neuronal morphology indicated in asterisks. **D-h**, Additional genes scoring differently in Ascl1 iN vs iT (**d**) and Ngn2 iN vs iNSC, that were validated using FACS (**f**) and immunostaining for neuronal marker Tubb3 (**g-h**) and iT – KRT8, CDX2, for Ascl1 (**g**) and iNSC – SOX2, PAX6, for Ngn2(**h**). Bar plots in **f)** shows mean of n=3 biologically independent samples ± SD. P values, indicated above, were determined by one-way ANOVA followed by Dunnett's multiple comparison test (two-sided) using Ctrl ratio as a control. "n.s." not significant, "*" < 0.05, "**" < 0.01, "***" < 0.001.

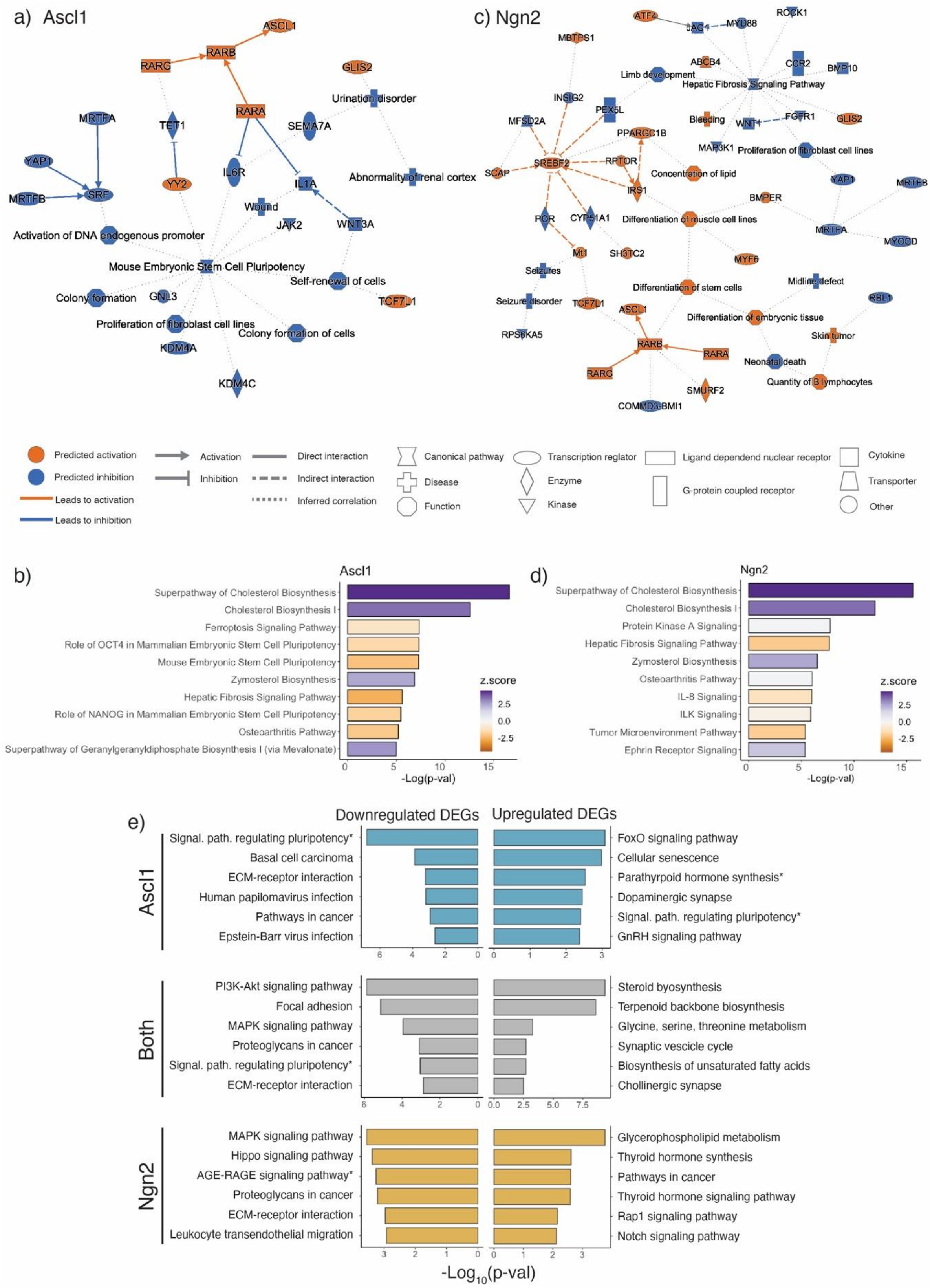
a) Ascl1
ASCL1
RARB
RARG
GLIS2
RARA
Urination disorder
MRTFA
TET1
SEMA7A
YAP1
IL6R
IL1A
Abnormality of renal cortex
SRF
YY2
MRTFB
Wound
JAK2
WNT3A
Activation of DNA endogenous promoter
Mouse Embryonic Stem Cell Pluripotency
Self-renewal of cells
Colony formation
GNL3
TCF7L1
Proliferation of fibroblast cell lines
Colony formation of cells
KDM1A
KDM4C
c) Ngn2
ATF4
JAG1
MYD88
ROCK1
MBTPS1
ABCB4
CCR2
BMP10
Limb development
Hepatic Fibrosis Signaling Pathway
INSIG2
PEX5L
MFSD2A
Bleeding
WNT1
FGFR1
GLIS2
PPARGC1B
MAP3K1
Proliferation of fibroblast cell lines
SCAP
SREBF2
RPTOR
Concentration of lipid
IRS1
YAP1
MRTFB
BMPER
POR
CYP51A1
Differentiation of muscle cell lines
MRTFA
MYOCD
Mt1
SH3TC2
MYF6
Seizures
Differentiation of stem cells
Midline defect
Seizure disorder
TCF7L1
ASCL1
RBL1
Differentiation of embryonic tissue
RPS6KA5
Skin tumor
RARB
RARA
Neonatal death
RARG
Quantity of B lymphocytes
SMURF2
COMMD3-BMI1
Predicted activation
Predicted inhibition
Leads to activation
Leads to inhibition
Activation
Inhibition
Direct interaction
Indirect interaction
Inferred correlation
Canonical pathway
Disease
Function
Transcription reglator
Enzyme
Kinase
Ligand dependend nuclear receptor
G-protein coupled receptor
Cytokine
Transporter
Other
b)
Ascl1
Superpathway of Cholesterol Biosynthesis
Cholesterol Biosynthesis I
Ferroptosis Signaling Pathway
Role of OCT4 in Mammalian Embryonic Stem Cell Pluripotency
Mouse Embryonic Stem Cell Pluripotency
Zymosterol Biosynthesis
Hepatic Fibrosis Signaling Pathway
Role of NANOG in Mammalian Embryonic Stem Cell Pluripotency
Osteoarthritis Pathway
Superpathway of Geranylgeranyldiphosphate Biosynthesis I (via Mevalonate)
z.score
2.5
0.0
-2.5
0
5
10
15
-Log(p-val)
d)
Ngn2
Superpathway of Cholesterol Biosynthesis
Cholesterol Biosynthesis I
Protein Kinase A Signaling
Hepatic Fibrosis Signaling Pathway
Zymosterol Biosynthesis
Osteoarthritis Pathway
IL-8 Signaling
ILK Signaling
Tumor Microenvironment Pathway
Ephrin Receptor Signaling
z.score
2.5
0.0
-2.5
0
5
10
15
-Log(p-val)
e)
Downregulated DEGs
Upregulated DEGs
Ascl1
Signal. path. regulating pluripotency*
Basal cell carcinoma
ECM-receptor interaction
Human papilomavirus infection
Pathways in cancer
Epstein-Barr virus infection
FoxO signaling pathway
Cellular senescence
Parathyrpoid hormone synthesis*
Dopaminergic synapse
Signal. path. regulating pluripotency*
GnRH signaling pathway
6 4 2 0
0 1 2 3
Both
PI3K-Akt signaling pathway
Focal adhesion
MAPK signaling pathway
Proteoglycans in cancer
Signal. path. regulating pluripotency*
ECM-receptor interaction
Steroid byosynthesis
Terpenoid backbone biosynthesis
Glycine, serine, threonine metabolism
Synaptic vescicle cycle
Biosynthesis of unsaturated fatty acids
Chollinergic synapse
6 4 2 0
0.0 2.5 5.0 7.5
Ngn2
MAPK signaling pathway
Hippo signaling pathway
AGE-RAGE signaling pathway*
Proteoglycans in cancer
ECM-receptor interaction
Leukocyte transendothelial migration
Glycerophospholipid metabolism
Thyroid hormone synthesis
Pathways in cancer
Thyroid hormone signaling pathway
Rap1 signaling pathway
Notch signaling pathway
3 2 1 0
0 1 2 3
-Log10(p-val)

Supplementary Figure 10. Additional analysis Ascl1 and Ngn2 overexpression transcriptional response
a, Ingenuity pathway analysis of the differentially expressed (LFC > 1 and LFC < -1, FDR < 0.05) genes 1 day post induction by Ascl1 (Fig. 1i). **b**, Ingenuity canonical pathway enrichment analysis of (**a**). P values were determined using Fisher's exact test. **c**, Ingenuity pathway analysis of the differentially expressed (LFC > 1 and LFC < -1, FDR < 0.05) genes 1 day post induction by Ngn2 (Fig. 1i). **d**, Ingenuity canonical pathway enrichment analysis of (**c**). P values were determined using Fisher's exact test. **e**, Kegg pathway enrichment analysis of the gene subgroups of Fig. 3a. Fisher's exact test was used to calculate p values. Asterisks indicate shortening of terms Signaling pathways regulating pluripotency of stem cells; Parathyroid hormone synthesis, secretion and action; AGE-RAGE signaling pathway in diabetic complications.

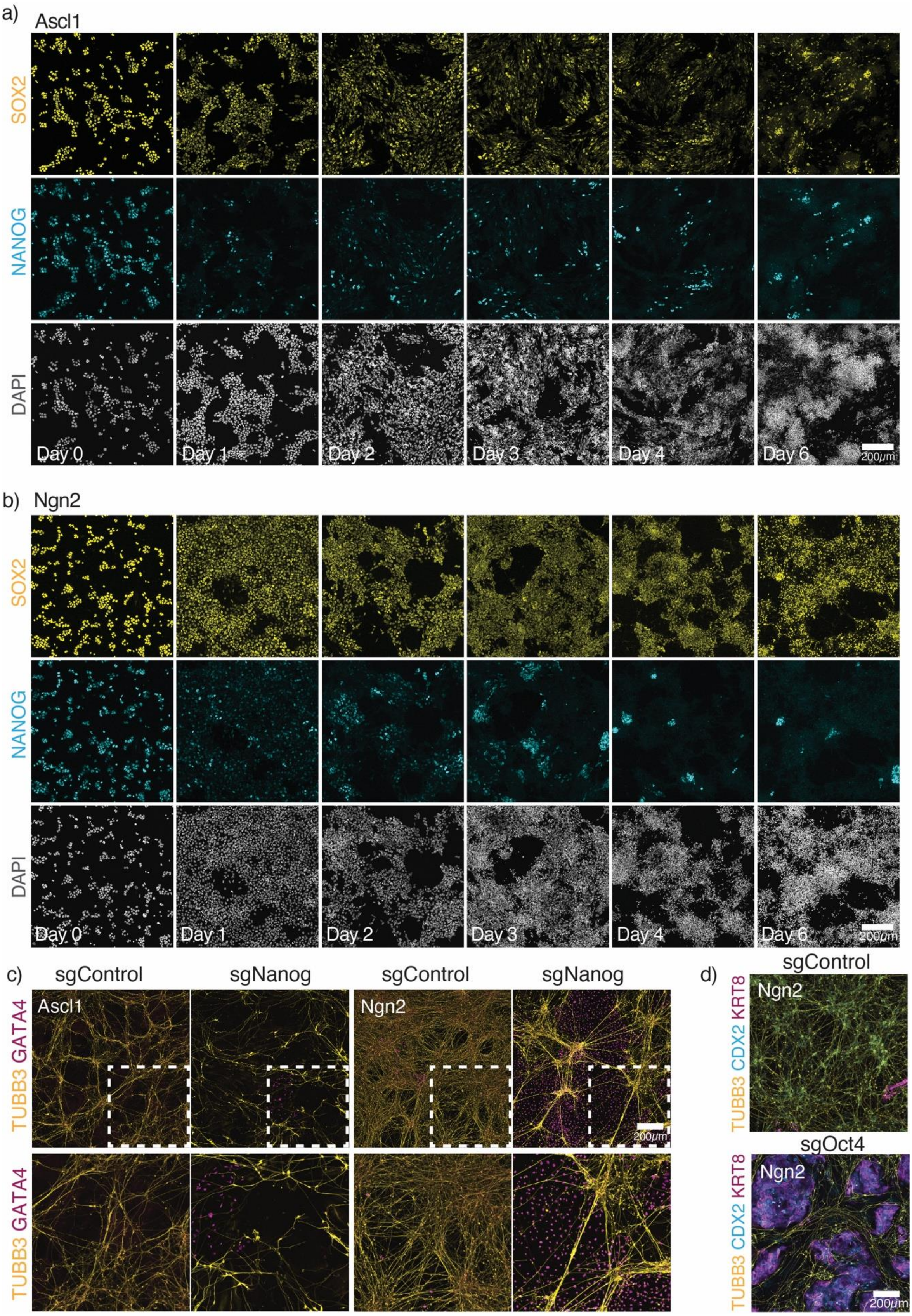

a)
Ascl1
SOX2
NANOG
DAPI
Day 0
Day 1
Day 2
Day 3
Day 4
Day 6
200μm
b)
Ngn2
SOX2
NANOG
DAPI
Day 0
Day 1
Day 2
Day 3
Day 4
Day 6
200μm
c)
sgControl
sgNanog
sgControl
sgNanog
TUBB3 GATA4
Ascl1
Ngn2
200μm
TUBB3 GATA4
d)
sgControl
TUBB3 CDX2 KRT8
Ngn2
sgOct4
TUBB3 CDX2 KRT8
Ngn2
200μm

Supplementary Figure 11. Comparison of the pluripotency network shutdown between Ascl1 and Ngn2 induction

a, Time course of the immunostained Ascl1 induced cells for the core pluripotency proteins SOX2 and NANOG. **b**, Time course of the immunostained Ngn2 induced cells for the core pluripotency proteins SOX2 and NANOG. **c**, Immunostaining for the neuronal TUBB3 and primitive endoderm marker GATA4 in the WT or with *Nanog* knockout cells 6 days post Ascl1 or Ngn2 induction. **d**, Immunostaining for the neuronal TUBB3 and trophoblast markers CDX2, KRT8 in the cells with *Pou5f1* (*Oct4)* knockout 6 days post Ngn2 induction.

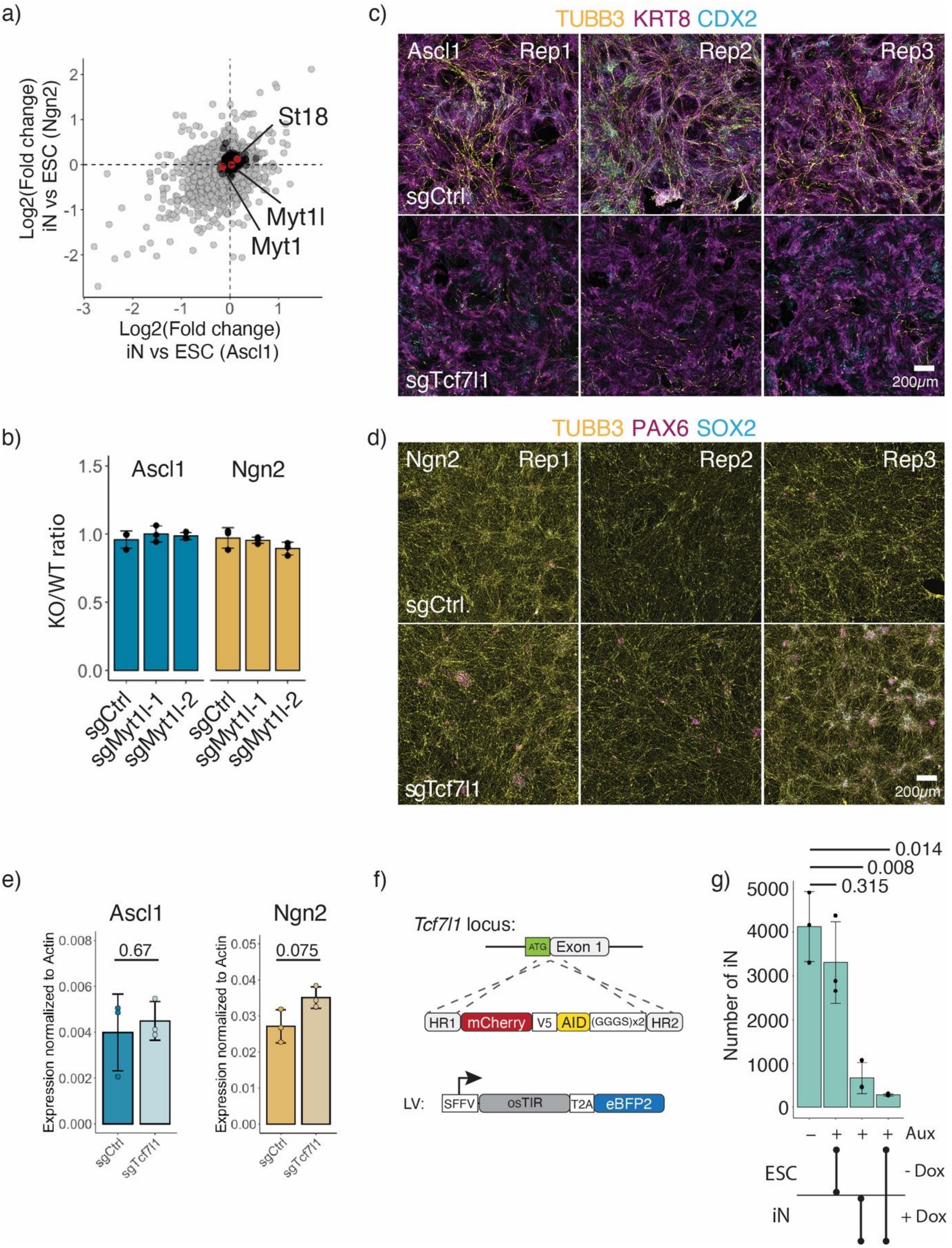
a)
Log2(Fold change) iN vs ESC (Ngn2)
Log2(Fold change) iN vs ESC (Ascl1)
St18
Myt1l
Myt1
b)
Ascl1
Ngn2
KO/WT ratio
sgCtrl
sgMyt1l-1
sgMyt1l-2
c)
TUBB3 KRT8 CDX2
Ascl1
Rep1
Rep2
Rep3
sgCtrl.
sgTcf7l1
200μm
d)
TUBB3 PAX6 SOX2
Ngn2
e)
Expression normalized to Actin
0.67
0.075
f)
Tcf7l1 locus:
ATG
Exon 1
HR1
mCherry
V5
AID
(GGGS)x2
HR2
LV:
SFFV
osTIR
T2A
eBFP2
g)
0.014
0.008
0.315
Number of iN
Aux
ESC
iN
- Dox
+ Dox

Supplementary Figure 12. Validation of CRISPR-Cas9 screen results in alternative systems

a, Effects of targeting *Myt1l*, *Myt1*, *St18* genes in CRISPR-Cas9 screen. Axis indicate Log_2(Fold change) of collapsed sgRNA abundance between ESC and iN. **b**, Validation of *Myt1l* knockout effect on iN directed differentiation. Experimental scheme same as Fig. 2f. Bar represents iN efficiency normalized to the efficiency of internal control cell population. Bar plot shows mean of n = 3 biologically independent samples with ± SD. **c-d**, Validation of *Tcf7l1* KO in E14 ESC polyclonally expressing Ascl1 or Ngn2. Immunostainings for neuronal TUBB3 and Trophoblast markers CDX2, KRT8 (Ascl1 (**a**)) or NSC markers PAX6, SOX2 (Ngn2 (**b**)). **e**, Expression of Ascl1 or Ngn2 cassette at day 1 post induction in WT or *Tcf7l1* knockout cells. Bar plot shows mean of n = 3 independent biological replicates with ± SD, p values of the two-sided Welch two sample t test indicated above. **f**, Scheme for N-terminally tagged Tcf7l1 with AID degron and lentiviral expression vector of osTIR. **g,** Efficiency of iN formation of Ascl1 expressing cells with AID-TCF7L1 (**d**) when TCF7L1 is degraded before, after induction or all the time. N = 3 biologically independent samples. Bar plot shows mean with ± SD, p values of the two-sided Welch two sample t test indicated above.

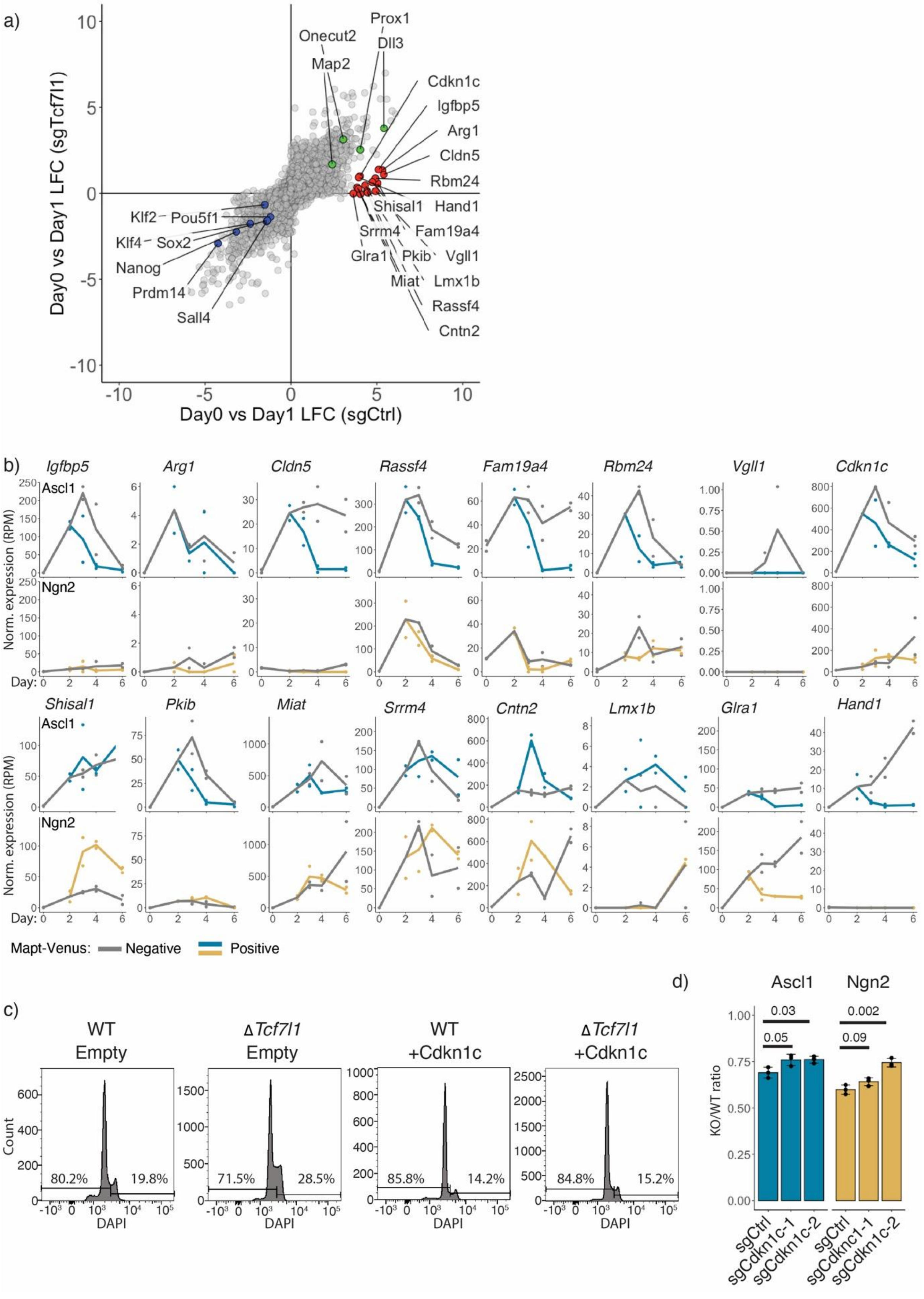

a)
Day0 vs Day1 LFC (sgTcf7l1)
Day0 vs Day1 LFC (sgCtrl)
Prox1
Onecut2
Dll3
Map2
Cdkn1c
Igfbp5
Arg1
Cldn5
Rbm24
Hand1
Shisal1
Fam19a4
Srrm4
Vgll1
Glra1
Pkib
Lmx1b
Miat
Rassf4
Cntn2
Klf2
Pou5f1
Klf4
Sox2
Nanog
Prdm14
Sall4
b)
Igfbp5
Arg1
Cldn5
Rassf4
Fam19a4
Rbm24
Vgll1
Cdkn1c
Ascl1
Ngn2
Norm. expression (RPM)
Day:
Shisal1
Pkib
Miat
Srrm4
Cntn2
Lmx1b
Glra1
Hand1
Mapt-Venus:
Negative
Positive
c)
WT
Empty
ΔTcf7l1
Empty
WT
+Cdkn1c
ΔTcf7l1
+Cdkn1c
Count
DAPI
80.2%
19.8%
71.5%
28.5%
85.8%
14.2%
84.8%
15.2%
d)
Ascl1
Ngn2
KO/WT ratio
0.03
0.05
0.002
0.09
sgCtrl
sgCdkn1c-1
sgCdkn1c-2
sgCtrl
sgCdknc1-1
sgCdkn1c-2

Supplementary Figure 13. Analysis of differentially expressed genes between WT and Tcf7l1 knockout cells.
a, Scatter plot comparing gene expression changes of Ascl1 induction at day 1 post between WT and Tcf7l1 knockout cells. Highlighted circles show genes upregulated in WT and not in *Tcf7l1* KO cells (red), example genes that are neuronal markers (green), core pluripotency genes (blue). N = 3. **b**, Expression over time (Fig. 1a. RNAseq data) of differentially upregulated genes (**(a)** – WT versus *Tcf7l1* KO, red circles). **c**, FACS analysis of cell cycle by DAPI staining at Day 3 between WT and *Tcf7l1* knockouts with or without expression of Cdkn1c. Percentages indicates cells in G1 versus other cell cycle stages. **d**, Effects of *Cdkn1c* knockout on iN formation. Bar represents iN efficiency normalized to the efficiency of internal control cell population. Experiment performed as a competition assay like in Fig. 2f. Normalized efficiency = (% of neurons in KO / % of neurons in WT). N = 3 biologically independent samples. Bar plot shows mean with ± SD, p values of the two-sided Welch two sample t test indicated above.

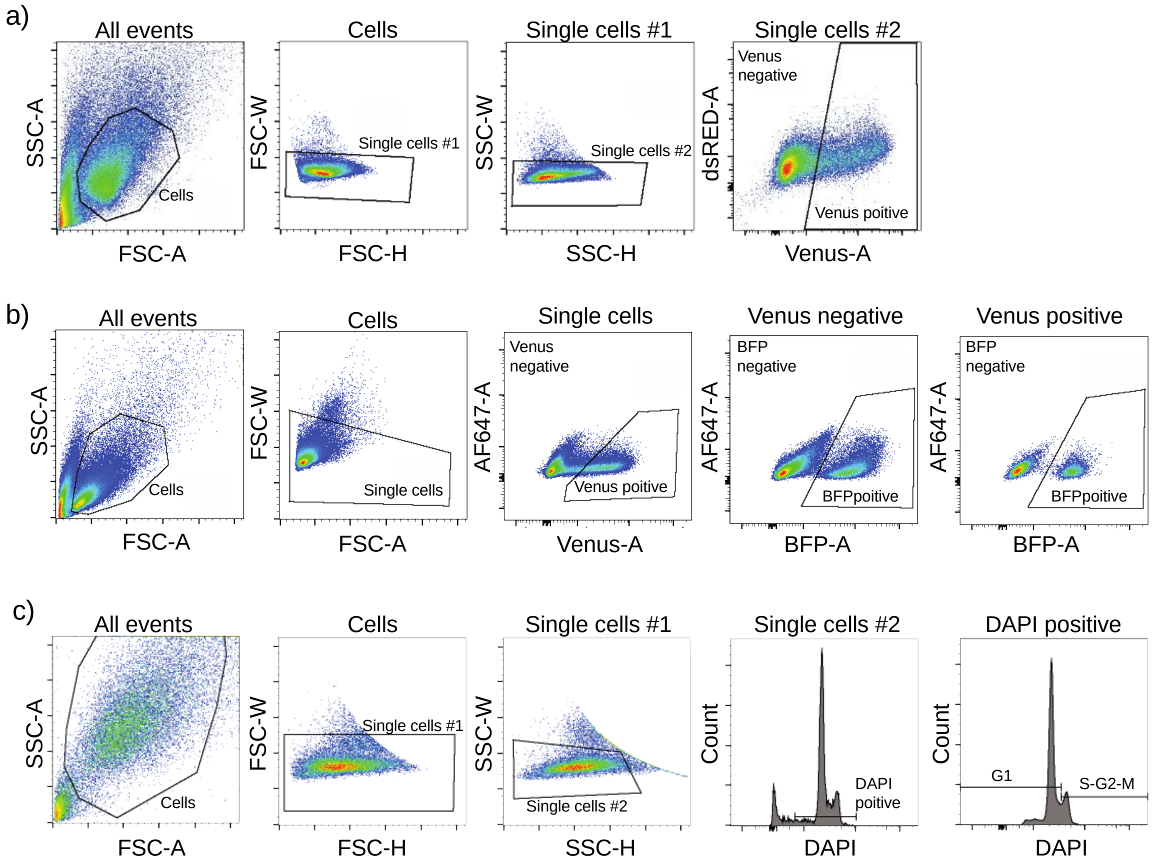

Supplementary Figure 14. FACS gating strategies

a, Representative FACS gating strategy to assess *Mapt*-Venus reporter in Fig. 3g-i, 4f, Supplement Fig. 2j, 4e, 8h, 9a, 12g. dsRED channel was used for assessing autofluorescence. **b**, Gating strategy used in competition assays in Fig. 2g, Supplement Fig. 9f, 12b, 13d. Alexa Fluor 647 (AF647) channel was used for measuring autofluorescence. **c,** Gating strategy for the supplement fig. 13c. Signal peak parameters are as follows: A – area, H – height, W – width.

Supplementary Table 1. Primers used for the sgRNA cloning.

sgRNA	Forward	Reverse
Ctrl	CACCGGAGTCGTTTTACCCGCCGC	AAACGCGGCGGGTAAAACGACTCC
Tcf7l1	CACCGCCTTCGGCGAAATAGTCGCG	AAACCGCGACTATTTCGCCGAAGGC
Sqle	CACCGAGTGTCGAATCAACACCAGA	AAACTCTGGTGTTGATTCGACACTC
Apc	CACCGGTACACCTGCTGAATACGAG	AAACCTCGTATTCAGCAGGTGTACC
Ptpn2	CACCGAAGAAGTTACATCTTAACAC	AAACGTGTTAAGATGTAACTTCTTC
Strap	CACCGGGCAAGCCCATGCTCCGCCA	AAACTGGCGGAGCATGGGCTTGCCC
Cnot8	CACCGTGTAGCTGAGGACAATCTCA	AAACTGAGATTGTCCTCAGCTACAC
Ccnk	CACCGTACCTTCCAGAACGTCAACA	AAACTGTTGACGTTCTGGAAGGTAC
Cdk13	CACCGATAAGTCGAGTGCAGCTGCG	AAACCGCAGCTGCACTCGACTTATC
Setd1b	CACCGCATGGGCAACATTATCCACG	AAACCGTGGATAATGTTGCCCATGC
Nanog_1	CACCGTCTGAACCTGAGCTATAAGC	AAACGCTTATAGCTCAGGTTCAGAC
Nanog_2	CACCGTAAGCAAGAATAGTTCTC	AAACGAGAACTATTCTTGCTTAC
Pou5f1_1	CACCGATCACCTTGGGGTACACCC	AAACGGGTGTACCCCAAGGTGATC
Pou5f1_2	CACCGCCCAGGGTGAGCCCCACGT	AAACACGTGGGGCTCACCCTGGGC
Sox2_1	CACCGCAGGGCGCTGACGTCGTAG	AAACCTACGACGTCAGCGCCCTGC
Sox2_2	CACCGAGCCCAGCGCCATACCGG	AAACCCGGTATGGCGCTGGGCTC
Cdkn1c_1	CACCGCTACGCGCTATCACTGGGA	AAACTCCCAGTGATAGCGCGTAGC
Cdkn1c_2	CACCGACAGCCACGGCCACCGG	AAACCCGGTGGCCGTGGCTGTC
Myt1l_1	CACCGATGGGTCAGGACACGTCAG	AAACCTGACGTGTCCTGACCCATC
Myt1l_2	CACCGAAGAAAGACGGTATCCAG	AAACCTGGATACCGTCTTTCTTC
Mapt_C-terminus_tag	CACCGGCCAAGCAGGGTTTGTGATC	AAACGATCACAAACCCTGCTTGGCC
Tcf7l1_N-terminus_tag	CACCGCCACCGAGCTGGGGCATGGT	AAACACCATGCCCCAGCTCGGTGGC

Supplementary Table 2. List of antibodies used.

Antibody	Host species	Producer	Catalogue #	Clone number	IF dilution
anti-Tubb3	Mouse	Sigma	T8660	SDL.3D10	1:500
anti-Tubb3	Rabbit	Biolegend/Covance	PRB-435P	-	1:500
anti-Map2	Rabbit	Abcam	ab32454	-	1:500
anti-Sox2	Rat	Invitrogen (eBioscience)	14-9811-80	Btjce	1:500
anti-Oct4	Rabbit	Abcam	ab19857	-	1:500
anti-Nanog	Rabbit	Abcam	ab80892	-	1:500
anti-Pax6	Rabbit	Covance	PRB-278P	-	1:200
anti-Krt8	Rat	DSHB	AB 531826	-	1:200
anti-Cdx2	Rabbit	Abcam	ab76541	EPR2764Y	1:400
anti-Nestin	Mouse	Merk	MAB353	rat-401	1:200
anti-Gata4	Rat	Invitrogen	14998082	eBioEvan	1:500
anti-Cdkn1c	Rabbit	Abcam	ab75974	-	1:250
anti-Mki67	Rat	Invitrogen (eBioscience)	14-5698-82	SolA15	1:200
anti-Ascl1	Mouse	Invitrogen (eBioscience)	14-5794-82	24B72D11	1:200
anti-Tpbpa	Rabbit	Abcam	ab104401		1:200
anti-Flag	Mouse	Sigma	F1804	M2	5ug/ChIP sample
anti-Mouse-488	Goat	Invitrogen	A11029	-	1:1000
anti-Rabbit-488	Goat	Invitrogen	A11034	-	1:1000
anti-Rat-488	Goat	Invitrogen	A11006	-	1:1000
anti-Mouse-568	Goat	Invitrogen	A11031	-	1:1000
anti-Rabbit-568	Goat	Invitrogen	A11036	-	1:1000
anti-Mouse-647	Goat	Invitrogen	A21247	-	1:1000
anti-Rabbit-647	Goat	Invitrogen	A21236	-	1:1000
anti-Rat-647	Goat	Invitrogen	A21245	-	1:1000

Supplementary Table 3. qPCR primers used.

Gene	Forward	Reverse
Nanog	GCCTCCAGCAGATGCAAGAAC	CTGGTGCTGAGCCCTTCTG
Pou5f1	GAGTCCCAGGACATGAAAGCC	CAGATGGTGGTCTGGCTGAAC
Sox2	CATGAGAGCAAGTACTGGCAAG	CCAACGATATCAACCTGCATGG
Acta	GCTGTATTCCCCTCCATCGTG	CACGGTTGGCCTTAGGGTTCAG
Ngn2-Mwcassette	GGCCCCGAATTCGCCACCAT	AGCTCCTCGTCCTCCTCCTC
Ascl1-Mwcassete	CCGAATTCGCTAGCCACCAT	AAGAAGCAGGCTGCGGG
