# Supplementary material for: Ascl1 and Ngn2 convert mouse embryonic stem cells to neurons via functionally distinct paths
Source: Nat Commun. 2023 Sep 2;14:5341. doi: 10.1038/s41467-023-40803-y (PMC10475046; doi:10.1038/s41467-023-40803-y)
Supplement: Supplementary file 2 — Reporting Summary [file 41467_2023_40803_MOESM2_ESM.pdf]

nature portfolio

Corresponding author(s): Gintautas Vainorius
Dr. Ulrich Elling

Last updated by author(s): Jul 15, 2023

Reporting Summary

Nature Portfolio wishes to improve the reproducibility of the work that we publish. This form provides structure for consistency and transparency in reporting. For further information on Nature Portfolio policies, see our Editorial Policies and the Editorial Policy Checklist.

Statistics

For all statistical analyses, confirm that the following items are present in the figure legend, table legend, main text, or Methods section.

n/a	Confirmed	
☐	☒	The exact sample size (*n*) for each experimental group/condition, given as a discrete number and unit of measurement
☐	☒	A statement on whether measurements were taken from distinct samples or whether the same sample was measured repeatedly
☐	☒	The statistical test(s) used AND whether they are one- or two-sided<br>*Only common tests should be described solely by name; describe more complex techniques in the Methods section.*
☒	☐	A description of all covariates tested
☐	☒	A description of any assumptions or corrections, such as tests of normality and adjustment for multiple comparisons
☐	☒	A full description of the statistical parameters including central tendency (e.g. means) or other basic estimates (e.g. regression coefficient) AND variation (e.g. standard deviation) or associated estimates of uncertainty (e.g. confidence intervals)
☐	☒	For null hypothesis testing, the test statistic (e.g. *F*, *t*, *r*) with confidence intervals, effect sizes, degrees of freedom and *P* value noted<br>*Give P values as exact values whenever suitable.*
☒	☐	For Bayesian analysis, information on the choice of priors and Markov chain Monte Carlo settings
☒	☐	For hierarchical and complex designs, identification of the appropriate level for tests and full reporting of outcomes
☒	☐	Estimates of effect sizes (e.g. Cohen's *d*, Pearson's *r*), indicating how they were calculated

Our web collection on statistics for biologists contains articles on many of the points above.

Software and code

Policy information about availability of computer code

Data collection	FACS data was obtained on FACS LSR II Fortessa (BD Bioscience) and iQue Screener PLUS (IntelliCyt®).<br>Confocal images were obtained on point laser scanning Zeiss AxioObserverZ1 LSM800 microcope.<br>NGS libraries were sequenced on Illumina HiSeq2500 and NextSeq550 machines.
Data analysis	FACS data analysis: Flowjo (v10.8.1), BD FACSDiva (v8.0), ForeCyt (v6.2.6752)<br>IF image analysis: ImageJ/Fiji (v1.53c), ZEN Black (v.2.3) or Zen Blue(v3.1)<br>Data processing, statistical analysis and visualization: RStudio with R(v4.1.0), ggplot2 (v3.3.6).<br>RNAseq analysis: read processing was done with BBDuk (v38.06), bowtie2 (v2.3.4.1), STAR (v2.6.0.c), featureCounts (v1.6.2); analysis was done in R using Deseq2(v1.32.0) package.<br>Gene regulatory network analysis: Cytoscape(v3.8.0), Ingenuity<br>ChIPseq analysis: read processing was done with trim-galore v0.4.4, BWA MEM (v0.7.17), Pircard MarkDuplicates (v2.23.4), deeptools (v3.5.0), MACS2 (v2.1.1), bedtools (v.2.27.1); analysis and vizualization was done in R using Diffbind (v.3.2.7), karyoploteR(v1.18.0) packages, MEME (v5.1.1)<br>CRISPR-Cas9 screens analysis: read processing was done with samtools (v1.9), fastx-toolkit(v0.0.14), bowtie(v1.1.2), python (v3.6.6); analysis and visualization was done using Mageck (v0.5.4) and R(v4.1.0).<br>Only standard bioinformatic code was used for the data analysis and is available on request.

For manuscripts utilizing custom algorithms or software that are central to the research but not yet described in published literature, software must be made available to editors and reviewers. We strongly encourage code deposition in a community repository (e.g. GitHub). See the Nature Portfolio guidelines for submitting code & software for further information.

Data

Policy information about availability of data

All manuscripts must include a data availability statement. This statement should provide the following information, where applicable:

- Accession codes, unique identifiers, or web links for publicly available datasets
- A description of any restrictions on data availability
- For clinical datasets or third party data, please ensure that the statement adheres to our policy

Raw NGS data produced in this study is deposited at Gene Expression Omnibus (GEO) database under super series accession number GSE206872: ChIP-seq (GSE206869), RNA-seq (GSE206870), CRISPR-Cas9 screen (GSE206871). RNA-seq and ChIP-seq reads were mapped to the GRCm38/mm10 assembly obtained from Mus musculus Ensembl release 94.

Human research participants

Policy information about studies involving human research participants and Sex and Gender in Research.

Reporting on sex and gender	N/A
Population characteristics	N/A
Recruitment	N/A
Ethics oversight	N/A

Note that full information on the approval of the study protocol must also be provided in the manuscript.

Field-specific reporting

Please select the one below that is the best fit for your research. If you are not sure, read the appropriate sections before making your selection.

☒ Life sciences ☐ Behavioural & social sciences ☐ Ecological, evolutionary & environmental sciences

For a reference copy of the document with all sections, see nature.com/documents/nr-reporting-summary-flat.pdf

Life sciences study design

All studies must disclose on these points even when the disclosure is negative.

Sample size	Complexity of the CRISPR-Cas9 screen was kept above 500 cells per sgRNAs during all the stages of the experiment.<br>No sample size calculation was performed otherwise and sample size was determined based on standard protocols in the field allowing estimation of variability of the results.
Data exclusions	In Fig. 2g screen validation experiment, a replicate was excluded for Setd1b due to inefficient number of cells and resulting poor sample quantification. No key conclusions were drawn based on this sample.
Replication	ChIPseq experiments contained two biological replicates with two technical replicates introduced by splitting sheared chromatin before incubation with antibody. RNAseq day 0 to day 6 time course (Fig. 1) was performed with two replicates, while early transcriptome analysis by RNAseq (Fig. 1 and Fig. 4) was performed in triplicate. CRISPR-Cas9 screens were carried our once (Fig. 2). Forward validations were carried out in triplicates and to ensure validity of the results, independent experiments with different readout were carrier out (e.g. FACS and IF). All replicates showed similar results.
Randomization	No randomization has been used as the cells were grown under identical conditions.
Blinding	No blinding was done since data were not analyzed subjectively.

Reporting for specific materials, systems and methods

We require information from authors about some types of materials, experimental systems and methods used in many studies. Here, indicate whether each material, system or method listed is relevant to your study. If you are not sure if a list item applies to your research, read the appropriate section before selecting a response.

Materials & experimental systems

n/a	Involved in the study
☐	☒ Antibodies
☐	☒ Eukaryotic cell lines
☒	☐ Palaeontology and archaeology
☒	☐ Animals and other organisms
☒	☐ Clinical data
☒	☐ Dual use research of concern

Methods

n/a	Involved in the study
☐	☒ ChIP-seq
☐	☒ Flow cytometry
☒	☐ MRI-based neuroimaging

Antibodies

Antibodies used

anti-Tubb3, Mouse, Sigma, T8660, clone SDL.3D10
anti-Tubb3, Rabbit, Biolegend/Covance, PRB-435P
anti-Map2, Rabbit, Abcam, ab32454
anti-Sox2, Rat, Invitrogen (eBioscience), 14-9811-80, clone Btjce
anti-Oct4, Rabbit, Abcam, ab19857
anti-Nanog, Rabbit, Abcam, ab80892
anti-Pax6, Rabbit, Covance, PRB-278P
anti-Krt8/TROMA-I, Rat, DSHB, AB 531826
anti-Cdx2, Rabbit, Abcam, ab76541, clone EPR2764Y
anti-Nestin, Mouse, Merk, MAB353, clone rat-401
anti-Gata4, Rat, Invitrogen, 14998082, clone eBioEvan
anti-Cdkn1c, Rabbit, Abcam, ab75974
anti-Mki67, Rat, Invitrogen (eBioscience), 14-5698-82, clone SolA15
anti-Ascl1, Mouse, Invitrogen (eBioscience), 14-5794-82, clone 24B72D11
anti-Tpbpa, Rabbit, Abcam, ab104401
anti-Flag, Mouse, Sigma, F1804, clone M2
anti-Mouse-488, Goat, Invitrogen, A11029
anti-Rabbit-488, Goat, Invitrogen, A11034
anti-Rat-488, Goat, Invitrogen, A11006
anti-Mouse-568, Goat, Invitrogen, A11031
anti-Rabbit-568, Goat, Invitrogen, A11036
anti-Mouse-647, Goat, Invitrogen, A21247
anti-Rabbit-647, Goat, Invitrogen, A21236
anti-Rat-647, Goat, Invitrogen, A21245

Validation

Antibodies used are from commercial sources and were previously used in published studies.
Concentration of antibodies were determined based on information provided by the manufacturer or other studies, as well as performing an antibody titration series. Cells not expressing antigen and samples without primary antibody during staining protocol were used as a negative controls to assess antibody specificity and background fluorescence.

Eukaryotic cell lines

Policy information about cell lines and Sex and Gender in Research

Cell line source(s)

Mouse embryonic stem cells (ESC) E14 (RRID:CVCL_9108)
Mouse embryonic fibroblasts (MEF, feeders) isolated from the E13.5 stage embryo of the DR4 mouse strain (RRID:IMSR_JAX:003208)

Authentication

None of the cell lines were authenticated by SNP analysis, yet cell lines remained visually identifyable throughout the study based on the ability to transdifferentiate into neurons.

Mycoplasma contamination

All cell lines were tested every 2 weeks and no Mycoplasma contamination was detected during the period of the study.

Commonly misidentified lines
(See ICLAC register)

Not used in this study

ChIP-seq

Data deposition

☒ Confirm that both raw and final processed data have been deposited in a public database such as GEO.

☒ Confirm that you have deposited or provided access to graph files (e.g. BED files) for the called peaks.

Data access links
May remain private before publication.

ChIP-seq raw NGS data is deposited at Gene Expression Omnibus (GEO) database under accession number GSE206869

Files in database submission

Accession_number Upload_name File_name
GSM6266261 Ascl1 Rep1.1 Ascl1_Rep1_1.fastq.gz
GSM6266262 Ascl1 Rep1.2 Ascl1_Rep1_2.fastq.gz
GSM6266263 Ascl1 Rep1.Input Ascl1_Rep1_Input.fastq.gz
GSM6266264 Ascl1 Rep2.1 Ascl1_Rep2_1.fastq.gz
GSM6266265 Ascl1 Rep2.2 Ascl1_Rep2_2.fastq.gz
GSM6266266 Ascl1 Rep2.Input Ascl1_Rep2_Input.fastq.gz
GSM6266267 Ngn2 Rep1.1 Ngn2_Rep1_1.fastq.gz
GSM6266268 Ngn2 Rep1.2 Ngn2_Rep1_2.fastq.gz
GSM6266269 Ngn2 Rep1.Input Ngn2_Rep1_Input.fastq.gz
GSM6266270 Ngn2 Rep2.1 Ngn2_Rep2_1.fastq.gz
GSM6266271 Ngn2 Rep2.2 Ngn2_Rep2_2.fastq.gz
GSM6266272 Ngn2 Rep2.Input Ngn2_Rep2_Input.fastq.gz
Processed data files:
Ascl1_Rep1_1.dedup.sorted.bam.bw
Ascl1_Rep1_2.dedup.sorted.bam.bw
Ascl1_Rep1_Input.dedup.sorted.bam.bw
Ascl1_Rep2_1.dedup.sorted.bam.bw
Ascl1_Rep2_2.dedup.sorted.bam.bw
Ascl1_Rep2_Input.dedup.sorted.bam.bw
Ngn2_Rep1_1.dedup.sorted.bam.bw
Ngn2_Rep1_2.dedup.sorted.bam.bw
Ngn2_Rep1_Input.dedup.sorted.bam.bw
Ngn2_Rep2_1.dedup.sorted.bam.bw
Ngn2_Rep2_2.dedup.sorted.bam.bw
Ngn2_Rep2_Input.dedup.sorted.bam.bw

Genome browser session
(e.g. UCSC)

https://data.bioinfo.vbc.ac.at/elling.grp/GSE206869/bw/Ascl1_Rep1_1.dedup.sorted.bam.bw
https://data.bioinfo.vbc.ac.at/elling.grp/GSE206869/bw/Ascl1_Rep1_2.dedup.sorted.bam.bw
https://data.bioinfo.vbc.ac.at/elling.grp/GSE206869/bw/Ascl1_Rep1_Input.dedup.sorted.bam.bw
https://data.bioinfo.vbc.ac.at/elling.grp/GSE206869/bw/Ascl1_Rep2_1.dedup.sorted.bam.bw
https://data.bioinfo.vbc.ac.at/elling.grp/GSE206869/bw/Ascl1_Rep2_2.dedup.sorted.bam.bw
https://data.bioinfo.vbc.ac.at/elling.grp/GSE206869/bw/Ascl1_Rep2_Input.dedup.sorted.bam.bw
https://data.bioinfo.vbc.ac.at/elling.grp/GSE206869/bw/Ngn2_Rep1_1.dedup.sorted.bam.bw
https://data.bioinfo.vbc.ac.at/elling.grp/GSE206869/bw/Ngn2_Rep1_2.dedup.sorted.bam.bw
https://data.bioinfo.vbc.ac.at/elling.grp/GSE206869/bw/Ngn2_Rep1_Input.dedup.sorted.bam.bw
https://data.bioinfo.vbc.ac.at/elling.grp/GSE206869/bw/Ngn2_Rep2_1.dedup.sorted.bam.bw
https://data.bioinfo.vbc.ac.at/elling.grp/GSE206869/bw/Ngn2_Rep2_2.dedup.sorted.bam.bw
https://data.bioinfo.vbc.ac.at/elling.grp/GSE206869/bw/Ngn2_Rep2_Input.dedup.sorted.bam.bw

Methodology

Replicates

ChIP-seq was performed in 2 replicates defined by independently derived ESC clones containing TetO-Flag-Ascl1-T2A-Puro or TetO-Flag-Ngn2-T2A-Puro. Before chromatin incubation with antibody, samples were split into two additional replicates.

Sequencing depth

All samples were sequenced as single-end 50mers
Sample Total Aligned
Ascl1_Rep1_1 29,826,937 29,069,058
Ascl1_Rep1_2 20,536,895 18,993,968
Ascl1_Rep1_Input 34,855,408 34,483,867
Ascl1_Rep2_1 42,912,586 41,744,738
Ascl1_Rep2_2 39,150,222 38,561,476
Ascl1_Rep2_Input 42,209,353 41,777,003
Ngn2_Rep1_1 28,364,683 25,909,253
Ngn2_Rep1_2 14,196,095 13,770,376
Ngn2_Rep1_Input 35,867,905 35,506,521
Ngn2_Rep2_1 30,455,827 29,593,923
Ngn2_Rep2_2 31,213,042 29,911,019
Ngn2_Rep2_Input 39,579,309 39,150,424

Antibodies

anti-Flag, Mouse, Sigma, F1804
Clone: M2, LOT#: SLCD3524

Peak calling parameters

Peaks were called using MACS v2.1.1. with q-value cut-off of 0.01.
Input samples was used as a reference to calculate read enrichment in peaks.

Data quality	We observed high read mapping rates (> 92%). Complexity and overall data quality was assessed using Phantompeakqualtools, Deeptools plotFingerprint. Correlation between replicates (Spearman correlation > 0.87) was assessed using Deeptools plotCorrleation.<br>Consensus peakset for the downstream analysis was generated using R Diffbind (v.3.2.7) if peak is present in all 4 replicates.<br>Validity of data was ensured by inspecting known targets of Ascl1 and Ngn2.
Software	Read processing was done with trim-galore v0.4.4, BWA MEM (v0.7.17), Pircard MarkDuplicates (v2.23.4), deeptools (v3.5.0), MACS2 (v2.1.1), bedtools (v.2.27.1)<br>Analysis and vizualization was done in R using Diffbind (v.3.2.7), karyoploteR(v1.18.0) packages, MEME (v5.1.1)

Flow Cytometry

Plots

Confirm that:

- [x] The axis labels state the marker and fluorochrome used (e.g. CD4-FITC).
- [x] The axis scales are clearly visible. Include numbers along axes only for bottom left plot of group (a 'group' is an analysis of identical markers).
- [x] All plots are contour plots with outliers or pseudocolor plots.
- [x] A numerical value for number of cells or percentage (with statistics) is provided.

Methodology

Sample preparation	Cells were trypsinized, blocked with FBS containing medium and single cell suspension for analysis was prepared in tubes or 96 well format
Instrument	FACS LSR II Fortessa (BD Bioscience); iQue Screener PLUS (IntelliCyt®)
Software	For data acquisition: BD FACSDiva8 (BD Bioscience); ForeCyt® (IntelliCyt®)<br>For data analysis: FlowJo, ForeCyt®
Cell population abundance	Induced neurons (iN) were reported with endogenously tagged mapt-V5-P2A-Venus-T2A-PuroTK reporter. In the standard conditions, population consists of 20-30% of iN, calculated as a percentage of Venus positive cells in a population. Neuronal population can be enriched to near purity by addition of cytosine β-D-arabinofuranoside (AraC) and Puromycin 4 days post induction. Flow cytometry results were confirmed by immunofluorescence.
Gating strategy	Live cells were gated using FSC-Area/SSC-Area. Single cells were gated by FSC-Area/FSC-Width and SSC-Area/FSC-Width. Venus positive cells were gated using 488 nm blue laser for excitation and 530/30 nm filter for detection.

- [x] Tick this box to confirm that a figure exemplifying the gating strategy is provided in the Supplementary Information.
